# A Multiscale Model for the World's First Parasitic Disease Targeted for Eradication: Guinea Worm Disease

**DOI:** 10.1155/2017/1473287

**Published:** 2017-07-20

**Authors:** Rendani Netshikweta, Winston Garira

**Affiliations:** Modelling Health and Environmental Linkages Research Group (MHELRG), Department of Mathematics and Applied Mathematics, University of Venda, Private Bag X5050, Thohoyandou 0950, South Africa

## Abstract

Guinea worm disease (GWD) is both a neglected tropical disease and an environmentally driven infectious disease. Environmentally driven infectious diseases remain one of the biggest health threats for human welfare in developing countries and the threat is increased by the looming danger of climate change. In this paper we present a multiscale model of GWD that integrates the within-host scale and the between-host scale. The model is used to concurrently examine the interactions between the three organisms that are implicated in natural cases of GWD transmission, the copepod vector, the human host, and the protozoan worm parasite* (Dracunculus medinensis)*, and identify their epidemiological roles. The results of the study (through sensitivity analysis of *R*_0_) show that the most efficient elimination strategy for GWD at between-host scale is to give highest priority to copepod vector control by killing the copepods in drinking water (the intermediate host) by applying chemical treatments (e.g., temephos, an organophosphate). This strategy should be complemented by health education to ensure that greater numbers of individuals and communities adopt behavioural practices such as voluntary reporting of GWD cases, prevention of GWD patients from entering drinking water bodies, regular use of water from safe water sources, and, in the absence of such water sources, filtering or boiling water before drinking. Taking into account the fact that there is no drug or vaccine for GWD (interventions which operate at within-host scale), the results of our study show that the development of a drug that kills female worms at within-host scale would have the highest impact at this scale domain with possible population level benefits that include prevention of morbidity and prevention of transmission.

## 1. Introduction

Guinea worm disease (GWD), sometimes known as* Dracunculiasis* or dracontiasis [1], is a nematode infection transmitted to humans exclusively through contaminated drinking water. People become infected when they drink water contaminated with copepods or cyclopoids (tiny aquatic crustaceans) harbouring infective Dracunculus larvae also known as* Dracunculus medinensis*. The larvae of* Dracunculus medinensis* are released into the stomach, when the copepods are digested by the effect of the gastric juice and get killed by the acid environment. Although the disease has low mortality, its morbidity is considerably high causing huge economic losses and devastating disabilities [2]. There is no vaccine or drug for the disease. Our ability to eliminate GWD rests partly on gaining better insights into the functioning of the immune system, especially its interaction with Guinea worm parasite and partly on development of drugs to treat the disease together with implementation of preventive measures. Currently, the only therapy for GWD is to physically extract the worm from the human body. Humans are the sole definitive host for GWD parasite. Efforts to eradicate the disease are focused on preventive measures which include the following:

- *Parasite control in the physical water environment*. This may involve chlorination of drinking water, or boiling the water before drinking, or applying a larvicide, all of which have the effect of killing the parasite and thereby reduce parasite population in the physical water environment.
- *Parasite control within the human host*. This involves physically extracting the worm from the human body by rolling it over an ordinary stick or matchstick [1, 3] and ensuring that the patient receives care by cleaning and bandaging the wound until all the worms are extracted from the patient. This process may take up to two months to complete, as the worm can grow up to a meter in length and only 1-2 centimeters can be removed per day [4, 5].
- *Vector control*. This consists of killing the copepods in water (the intermediate host) by applying a chemical called temephos, an organophosphate, to unsafe drinking water sources every month during the transmission season, thus reducing vector population and reducing the chances of individuals contracting the disease [2, 6, 7]. The adult vector may also be removed from drinking water by filtering the water using a nylon cloth or by boiling the water.
- *Health education*. This is disseminated through poster, radio and television broadcast, village criers and markets, face-to-face communication (social mobilization and house-to-house visits) by health workers and volunteers to ensure that greater numbers of individuals and communities adopt behavioural practices aimed at preventing transmission of GWD [8]. These behavioural practices include voluntary reporting of GWD cases, prevention of GWD patients from entering drinking water bodies, regular use of water from safe water sources, and, in the absence of such water sources, filtering or boiling water before drinking [6].
- *Provision of safe water sources*. This involves providing safe drinking water supplies through protecting hand-dug wells and sinking deep bore wells, improving existing surface water sources by constructing barriers to prevent humans from entering water, and filtering the water through sand-filters [4].

To date, these preventive measures have reduced the incidence of GWD by over 99% [6], making GWD the most likely parasitic disease that will soon be eradicated without the use of any drug or vaccine. Most countries, including the whole of Asia, are now declared free from GWD and transmission of the disease is now limited to African countries, especially Sudan, Ghana, Mali, Niger, and Nigeria [8]. GWD is one of the neglected tropical diseases. It is also an environmentally driven infectious disease. Therefore, its transmission depends on the parasite's survival in the environment and finding new hosts (humans and copepod vectors) in order to replicate and sustain parasite population. Because this process is complex, it has hampered eradication efforts. During the parasite's movement through the environment to the human and copepod vector hosts, many environmental factors influence both the parasite's population and the vector population.

For infectious diseases, including environmentally driven infectious diseases such as GWD, mathematical models have a long history of being used to study their transmission and also to compare and evaluate the effectiveness and affordability of intervention strategies that can be used to control or eliminate them [9, 10]. Currently, the predominant focus of modelling of infectious diseases is centered on concepts of epidemiological modelling and immunological modelling being considered as separate disease processes even for the same infectious disease. In epidemiological or between-host modelling of infectious diseases, the focus is on studying of transmission of infectious diseases between hosts, be they animals or humans or even both in the case of multiple host infections. In the immunological or within-host modelling of infectious diseases, the focus is on studying the interaction of pathogen and the immune system together with other within-host processes in order to elucidate outcomes of infection within a single host [11, 12]. To the best of our knowledge there has been no mathematical model to study the multiscale nature of GWD transmission by integrating between-host scale and within-host scale disease processes. Such models are sometimes called immunoepidemiological models [13]. Most of the mathematical models that have been developed so far are focused on the study of GWD at the epidemiological scale [14–16]. The purpose of this study is to develop an immunoepidemiological model of GWD. Immunoepidemiological modelling of infectious diseases is the quantitative approach which assists in developing a systems approach to understanding infectious disease transmission dynamics with regard to the interdependences between epidemiological (between-host scale) and immunological (within-host scale) processes [17, 18]. The immunoepidemiological model of GWD presented in this paper is based on a modelling framework of the immunoepidemiology of environmentally driven infectious diseases developed recently by the authors [13]. This new and innovative immunoepidemiological modelling framework, while maintaining the limits of a mathematical model, offers a solid platform to bring the separate modelling efforts (immunological modelling and epidemiological modelling) that focus on different aspects of the disease processes together to cover a broad range of disease aspects and time-scales in an integrated systems approach. It bridges host, environmental, and parasitic disease phenomena using mathematical modelling of parasite-host-environment-vector interactions and epidemiology to illuminate the fundamental processes of disease transmission in changing environments. For GWD there are three distinct time-scales associated with its transmission cycle which are as follows.

- *The epidemiological time-scale*, which is associated with the infection between hosts (human and copepod vector hosts).
- *The within-host time-scale*, which is related to the replication and developmental stages of Guinea worm parasite within an individual human host and the individual copepod vector host.
- *The environmental time-scale*, which is associated with the abundance and survival of Guinea worm parasite population and vector population in the physical water environment.

In order to try and integrate these different processes and the associated time-scales of GWD, the immunoepidemiological model of GWD presented here incorporates the actual parasite load of the human host and copepod vector, rather than simply tracking the total number of infected humans. It also incorporates the various stages of the parasite's life cycle as well as the within-host effects such as the effect of gastric juice within an infected human host and describes how the life stages in the definitive human host, environment, and intermediate vector are interconnected with the parasite's life cycle through contact, establishment, and parasite fecundity. The paper is organized as follows. In Section 2 we present brief discussion of the life cycle of Guinea worm parasite and use this information to develop the immunoepidemiological model of GWD in the same section. In Sections 3, 4, and 5, we derive the analytical results associated with the immunoepidemiological model and show that the model is mathematically and epidemiologically well-posed. We also show the reciprocal influence between the within-host scale and between-host scale of GWD transmission dynamics. The results of the sensitivity analysis of the reproductive number are given in Section 6 while the numerical results of the model are presented in Section 7. The paper ends with conclusions in Section 8.

## 2. The Mathematical Model

**Figure 1:**
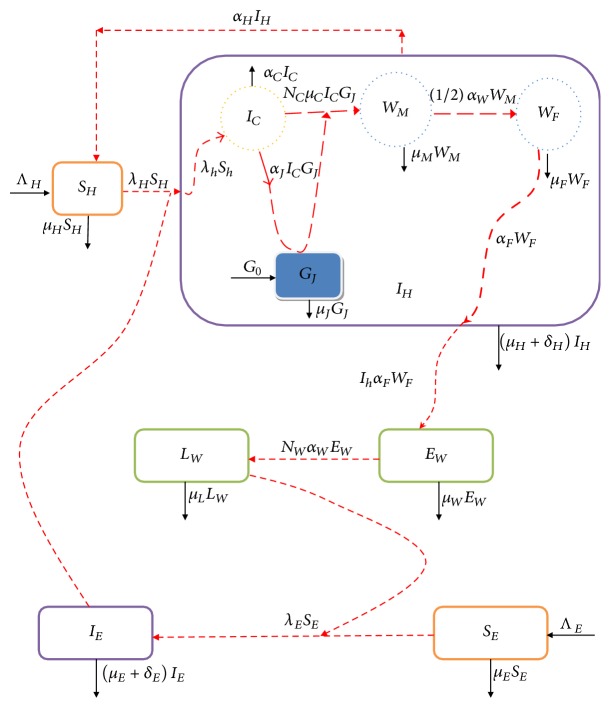
A conceptual diagram of the multiscale model of Guinea worm disease transmission dynamics.

We develop a multiscale model of Guinea worm disease that traces the parasite's life cycle of Guinea worm disease. The life cycle of GWD involves three different environments: physical water environment, biological human host environment, and biological copepod host environment. For more details on the life cycle of GWD see the published works [6, 19]. We only give a brief description in this section. The transmission cycle of Guinea worm disease begins when the human individual drinks contaminated water with copepods that are infected with Guinea mature worm larvae (L3 larvae). After ingestion, gastric juice in the human stomach kills the infected copepods and mature worm larvae are released. Then the released mature worm larvae penetrate the human stomach and intestinal wall and move to abdominal tissues where they grow and mate. After mating the male worms die soon and fertilized female worms migrate towards the skin surface (usually on the lower limbs or feet). After a year of infection, the fertilized female worm makes a blister on the infected individual's skin causing burning and itching, which forces an infected individual to immerse his or her feet into water (which is the only source of drinking water) to seek relief from pain. At that point the female worm emerges and releases thousands of worm eggs. The worm eggs then hatch Guinea worm larvae (L1 larvae stage) which are then consumed by copepods and take approximately two weeks to develop and become infective mature larvae (L3 larvae) within the copepods. Then ingestion of the infected copepods by human closes the life cycle. The multiscale model which we now present explicitly traces this life cycle of* Dracunculus medinensis* in three different environments, which are physical water environment, biological human environment, and biological copepod environment. The model flow diagram is shown in Figure 1.

The full multiscale model presented in this paper is based on monitoring the dynamics of ten populations at any time *t*, which are susceptible humans *S*_*H*_(*t*) and infected humans *I*_*H*_(*t*) in the behavioural human environment; infected copepods *I*_*C*_ in the human biological environment; mature Guinea worms *W*_*M*_(*t*) and fertilized female Guinea worms *W*_*F*_(*t*) in the biological human environment (within-host parasite dynamics); Guinea worm eggs *E*_*W*_(*t*) and Guinea worm larvae *L*_*W*_(*t*) in the physical water environment; susceptible copepods *S*_*E*_(*t*) and infected copepods *I*_*E*_(*t*) in the physical water environment; and gastric juice *G*_*J*_(*t*) in the human biological environment. We make the following assumptions for the model:

- There is no vertical transmission of the disease.
- The transmission of the disease in the human population is only through drinking contaminated water with infected copepods, *I*_*E*_(*t*), harbouring infective free-living pathogens (first-stage larvae), *L*_*W*_(*t*), in the physical water environment.
- For an infected individual, more than one Guinea worm can emerge simultaneously or sequentially over the course of weeks, depending on the number and intensity of infection the preceding year.
- Humans do not develop temporary or permanent immunity.
- Copepods do not recover from infection.
- The total population of humans and copepods is constant.
- Except for the effects of gastric juice in the stomach, there is no immune response in the human host.
- Copepods die in the human stomach due to the effects of gastric juice at a rate *α*_*C*_ before their larvae undergoes two molts in the copepod to become L3 larvae and therefore are nonviable and noninfectious larvae.

From the model flow diagram presented in Figure 1 and the assumptions that we have now made, we have the following system of ordinary differential equations as our multiscale model for GWD transmission dynamics:

(1) 1  dSHtdt=ΛH−λHtSHt−μHSHt+αHIHt,2  dIHtdt=λHtSHt−μH+δH+αHIHt,3  dICtdt=λhtSht−μCGJtICt−αCICt,4  dWMtdt=NCμCGJtICt−αM+μMWMt,5  dWFtdt=αM2WMt−μF+αFWFt,6  dGJtdt=G0+αJGJtICt−μJGJt,7  dEWtdt=αFWFtIht−μW+αWEWt,8  dLWtdt=NWαWEWt−μLLWt,9  dSEtdt=ΛE−λEtSEt−μESEt,10  dIEtdt=λEtSEt−μE+δEIEt,

where

(2) λHt=βHIEtP0+ϵIEt,λEt=βELWtL0+ϵLWt,Iht=IHt+1,Sht=SHt−1,λht=βHIEtP0+ϵIEtIHt+1.

Equations (1) and (2) of the model system (1) describe the evolution with time of susceptible and infected human hosts, respectively. At any time *t*, new susceptible humans are recruited at a constant rate Λ_*H*_ and we assume that the recruited humans are all susceptible. Susceptible individuals leave the susceptible class either through infection at rate *λ*_*H*_(*t*)*S*_*H*_(*t*) by drinking contaminated water with infected copepods to join infected group or through natural death at a rate *μ*_*H*_. The infected group is generated through infection when susceptible humans acquire the disease at a rate *λ*_*H*_(*t*)*S*_*H*_(*t*) through drinking water contaminated with copepods infected with* Dracunculus medinensis*. Infected humans leave the infected group either through recovery at a rate *α*_*H*_ to join the susceptible group or through natural death at a rate *μ*_*H*_, or through disease induced death at a rate *δ*_*H*_. Equation (3) of the model system (1) represents the evolution with time of infected copepods within an infected human host. The infected copepods within a human host are generated following uptake of infected copepods in the physical water environment through drinking contaminated water. In the human population, this uptake of infected copepods, which harbour Guinea worm larvae, is the transmission of Guinea worm parasite from the physical water environment to susceptible humans who become infected humans. Following the methodology described in [13] for modelling reinfection (superinfection) for environmentally transmitted infectious disease systems (because GWD and schistosomiasis are both water-borne and vector-borne infections), we model the average rate at which a single susceptible human host uptakes the infected copepods in the physical water environment through drinking contaminated water and becomes an infected human host by the expression

(3) $$\begin{array}{c} \lambda_{h}\left(\;t\;\right)S_{h}\left(\;t\;\right)=\frac{\lambda_{H}\left(t\right)\left[S_{H}\left(t\right)-1\right]}{\left[I_{H}\left(t\right)+1\right]}, \end{array}$$

where *λ*_*H*_(*t*), *S*_*H*_(*t*), and *I*_*H*_(*t*) are as defined previously. This is because, in our case, we define such a single infection by a single transition

(4) SHt,IHt,IEt⟶SHt−1,IHt+1,IEt.

Therefore, the average number of infected copepods, *I*_*C*_(*t*), within a single infected human host increases at a mean rate *λ*_*h*_(*t*)*S*_*h*_(*t*) and decreases through death due to digestion by human gastric acid at a rate *μ*_*C*_ after their larvae undergo two molts in the copepod to become L3 larvae and release viable and infectious larvae or naturally at a rate *α*_*C*_ before their larvae undergo two molts in the copepod to become L3 larvae and release nonviable and noninfectious larvae.

Equations (4–6) of the model system (1) represent changes with time of the average population of mature worms *W*_*M*_(*t*), fertilized female worms *W*_*F*_(*t*), and the amount of gastric acid *G*_*J*_(*t*) within a single infected human host, respectively. The average mature worm population *W*_*M*_(*t*) in a single infected human host is generated following the digestion of infected copepods in the human stomach by gastric acid and then mature worms are released. We assume that mature worms die naturally at a rate *μ*_*M*_ and they exit the human stomach to the abdominal tissues at a rate *α*_*M*_, where they grow and mate. The population of fertilized female worms, *W*_*F*_(*t*) within an infected human host, is generated following the developmental changes undergone by mature fertilized female worms. These developmental changes result in mature worms reaching sexual maturity and mating and all male worms die soon after mating. We assume that fertilized female worms die naturally at a rate *μ*_*F*_ and emerge out through an infected human individual's skin (usually the lower limbs) to release Guinea worm eggs into a water source at a rate *α*_*F*_, when an infected human comes into contact with water. The average amount of gastric acid inside a human stomach is generated following copepod vector induced proliferation at a rate *α*_*J*_*I*_*C*_(*t*), which is proportional to the density of infected copepods within an infected human host. We assume that the amount of gastric acid is also increased by the spontaneous production of gastric acid by the human body at a rate *G*_0_ and diluted or degraded at a rate *μ*_*J*_. Equation (7) of model system (1) describes the evolution with time of the Guinea worm eggs *E*_*W*_(*t*) in the physical water environment. We note that the population of Guinea worm eggs increases when each infected human host excretes eggs at a rate *α*_*F*_*W*_*F*_(*t*). Therefore the rate at which infected humans contaminate the physical water environment by excreting Guinea worm eggs is modelled by *α*_*F*_*W*_*F*_(*t*)*I*_*h*_(*t*). The last three equations of the model system (1) describe the evolution with time of Guinea worm larvae *L*_*W*_(*t*), susceptible copepods *S*_*E*_(*t*), and infected copepods *I*_*E*_(*t*) in the physical water environment, respectively. The population of Guinea worm larvae is generated through each egg hatching an average of *N*_*W*_ worms larvae with eggs hatching at an average rate of *α*_*W*_. Therefore the total Guinea worm larvae in the physical water environment are modelled by *N*_*W*_*α*_*W*_*E*_*W*_(*t*). We assume that worm larvae in the physical water environment die naturally at a constant rate *μ*_*L*_. Similar to human population, at any time *t*, new susceptible copepods are recruited at a constant Λ_*E*_. Susceptible copepods leave the susceptible group to join the infected copepods group through infection at a rate *λ*_*E*_(*t*)*S*_*E*_(*t*) when they consume first-stage Guinea worm larvae in the physical water environment. We assume that the population of copepods die naturally at a constant rate *μ*_*E*_ and further, we also assume that infected copepods have an additional mortality rate *δ*_*E*_ due to infection. The model state variables are summarized in Table 1.

## 3. Invariant Region of the Model

The model system (1) can be analysed in a region *Ω* ⊂ *ℝ*_+_^10^ of biological interest. Now assume that all parameters and state variables for model system (1) are positive for all *t* > 0 and further suppose that *G*_*J*_ is bounded above by *G*_0_/*μ*_*J*_. It can be shown that all solutions for the model system (1) with positive initial conditions remain bounded.

Letting *N*_*H*_ = *S*_*H*_ + *I*_*H*_ and adding (1) and (2) of model system (1) we obtain

(5) dSHdt+dIHdtdNHdt=ΛH−μHNH−δHIH≤ΛH−μHNH.

This implies that

(6) $$\begin{array}{c} \underset{t\to \infty }{lim}\sup\left(\;N_{H}\left(\;t\;\right)\;\right)\leq \frac{\Lambda_{H}}{\mu_{H}}. \end{array}$$

Similarly, letting *N*_*E*_ = *S*_*E*_ + *I*_*E*_ and adding (9) and (10) of model system (1) we obtain

(7) dSEdt+dIEdtdNEdt=ΛE−μHNE−δHIE≤ΛE−μHNE.

This also implies that

(8) $$\begin{array}{c} \underset{t\to \infty }{lim}\sup\left(\;N_{E}\left(\;t\;\right)\;\right)\leq \frac{\Lambda_{E}}{\mu_{E}}. \end{array}$$

Now considering the third equation of model system (1), given by

(9) dICdtλhSh−μCGJIC−δCIC=βHIESH−1P0+ϵIEIH+1−μCGJ+αCIC,

we obtain

(10) dICdt≤βHΛEΛH−μHP0μE+ϵΛEΛH+μH−1μJμCG0+αCμJIC.

This implies that

(11) limt→∞sup⁡ICt≤βHΛEΛH−μHP0μE+ϵΛEΛH+μHμJμCG0+δHμJ.

Using (6), (8), and (11) similar expression can be derived for the remaining model variables. Hence, all feasible solutions of the model system (1) are positive and enter a region defined by

(12) Ω=SH,IH,IC,WM,WF,GJ,EW,LW,SE,IE∈R+10:0≤SH+IH≤S1,  0≤SE+IE≤S2,  0≤IC≤S3,  0≤WM≤S4,  0≤WF≤S5,  0≤GJ≤S6,  0≤EW≤S7,  0≤LW≤S8,

which is positively invariant and attracting for all *t* > 0, where

(13) S1=ΛHμH,S2=ΛEμE,S3=μJμCG0+αCμJS9,S4=NCαCμCαM+μMμJμCG0+αCμJS9,S5=12αMαF+μFNCαCμCαM+μMμJμCG0+αCμJS9,S6=G0μJ,S7=12αFαW+μWαMαF+μFNCαCμCαM+μM·μJμCG0+αCμJS9,S8=12NWαWμLαFαW+μWαMαF+μF·NCαCμCαM+μMμJμCG0+αCμJS9,S9=βHΛCΛH−μHP0μC+ϵΛCΛH+μH.

Therefore it is sufficient to consider solutions of the model system (1) in *Ω*, since all solutions starting in *Ω* remain there for all *t* ≥ 0. Hence, the model system is mathematically and epidemiologically well-posed and it is sufficient to consider the dynamics of the flow generated by model system (1) in *Ω* whenever Λ_*H*_ > *μ*_*H*_. We shall assume in all that follows (unless stated otherwise) that Λ_*H*_ > *μ*_*H*_.

## 4. Determination of Disease-Free Equilibrium and Its Stability

To obtain the disease-free equilibrium point of system (1), we set the left-hand side of the equations equal to zero and further we assume that *I*_*H*_ = *I*_*C*_ = *W*_*H*_ = *W*_*H*_ = *E*_*W*_ = *L*_*W*_ = *I*_*E*_ = 0. This means that all the populations are free from the disease. Thus we get

(14) E0=SH0,IH0,IC0,WM0,WF0,GJ0,EW0,LW0,SE0,IE0,=ΛHμH,0,0,0,0,G0μJ,0,0,ΛEμE,0,

as the disease-free equilibrium of the model system (1).

### 4.1. The Basic Reproduction Number of the Model System (1)

The basic reproduction number of the system model (1) is calculated in this section using next generation operator approach described in [20]. Thus the model system (1) can also be written in the form

(15) dXdt=fX,Y,Z,dYdt=gX,Y,Z,dZdt=hX,Y,Z,

where

- *X* = (*S*_*H*_, *S*_*E*_, *G*_*J*_) represents all compartments of individuals who are not infected,
- *Y* = (*I*_*H*_, *I*_*C*_, *W*_*M*_, *W*_*F*_, *E*_*W*_) represents all compartments of infected individuals who are not capable of infecting others,
- *Z* = (*I*_*E*_, *L*_*W*_) represents all compartments of infected individuals who are capable of infecting others.

We also let the disease-free equilibrium of the model (1) be denoted by the following expression:

(16) $$\begin{array}{c} {\bar{U}}_{0}=\left(\;\frac{\Lambda_{H}}{\mu_{H}},0,0,0,0,\frac{G_{0}}{\mu_{J}},0,0,\frac{\Lambda_{E}}{\mu_{E}},0\;\right). \end{array}$$

Following [20], we let

(17) g~X∗,Z=g~1X∗,Z,g~2X∗,Z,g~3X∗,Z,g~4X∗,Z,g~5X∗,Z,

with

(18) g~1X∗,Z=βHΛHZ1μHμH+δH+αHP0+ϵIE,g~2X∗,Z=βHΛH−μHμJμH+δH+αHZ1μCG0+μJαCM11,g~3X∗,Z=NCαCβHΛH−μHG0μH+δH+αHZ1μM+αMμCG0+μJαCM11,g~4X∗,Z=αMNCαCβHΛH−μHG0μH+δH+αHZ12μF+αFμM+αMμCG0+μJαCM11,g~5X∗,Z=αFαMNCαCβHΛH−μHG0Z12μW+αWμF+αFμM+αMμCG0+μJαCμHP0+ϵIE,

where

(19) $$\begin{array}{c} M_{11}=\epsilon \beta_{H}\Lambda_{H}I_{E}+\mu_{H}\left(\;\mu_{H}+\delta_{H}+\mu_{H}\;\right)\left(\;P_{0}+\epsilon I_{E}\;\right). \end{array}$$

We deduce that

(20) $$\begin{array}{c} h\left(\;X,Y,Z\;\right)=\left(\;h_{1}\left(\;X,Y,Z\;\right),h_{2}\left(\;X,Y,Z\;\right)\;\right), \end{array}$$

with

(21) h1X,Y,ZλESE−μE+αEIE=βEΛEZ2μEL0+ϵZ2−μE+αEZ1,h2X,Y,ZNWαWEW−μLLW=KZ1P0+ϵZ1−μLZ2,

where

(22) K=αFαMNWαWNCαCβHΛH−μHG02μW+αWμF+αFμM+αMμCG0+μJαCμH.

*A* matrix

(23) $$\begin{array}{c} A=D_{Z}h\left(\;X^{*},\tilde{g}\left(\;X^{*},0\;\right),0\;\right)=\left[\begin{array}{cc} -\left(\mu_{E}+\alpha_{E}\right) & \frac{K}{P_{0}}\\ \frac{\beta_{E}\Lambda_{E}}{\mu_{E}L_{0}} & -\mu_{L} \end{array}\right] \end{array}$$

can be written in the form *A* = *M* − *D*, so that

(24) M=0KP0βEΛEμEL00,

(25) D=μE+αE00μL.

The basic reproductive number is the spectral radius (dominant eigenvalue) of the matrix *T* = *MD*^−1^. Hence, the basic reproduction number of the immumoepidemiological model (1) is expressed by the following quantity.

(26) R0=12·αMαM+μM·αFαF+μF·NCμCG0μCG0+μJαC·βHΛH−μHP0μH·NWαWαW+μWμLβEΛEμEμE+δEL0=R0BR0W,

with

(27) R0B=βHΛH−μHP0μH·NWαWαW+μWμLβEΛEμEμE+δEL0,

(28) R0W=12·αMαM+μM·αFαF+μF·NCμCG0μCG0+μJαC.

The expression, *R*_0*B*_, in (27) represents GWD's partial reproductive number associated with the between-host transmission of the disease while the expression, *R*_0*W*_, in (28) represents GWD's partial reproductive number associated with the within-host transmission of the disease. From the above two expressions in (27) and (28), respectively, we therefore make the following deductions.

- The epidemiological (between-host) transmission parameters such as the rate at which susceptible humans come into contact with water contaminated with infected copepods *β*_*H*_ (through drinking contaminated water with infected copepods) and the rate at which susceptible copepods come into contact with Guinea worm larvae *β*_*E*_; the supply rate of susceptible humans Λ_*H*_ and copepods Λ_*E*_ (through birth); the rate at which worms emerge from infected humans to contaminate the physical water environment *α*_*F*_, by laying eggs every time infected humans come into contact with water sources; the rate at which eggs in physical water environment hatch to produce worm larvae *N*_*W*_*α*_*W*_ all contribute to the transmission of Guinea worm disease. Therefore control measures such as reducing the rate at which infected human hosts visit water sources when an individual is infected, reducing contact rate between susceptible humans with contaminated water through educating the public, and treating water bodies with chemicals that kill worm eggs, worm larvae, and copepods may help to reduce the transmission risk of GWD.
- The immunological (within-host) transmission parameters such as the rate at which infected copepods within an infected human host release mature worms *N*_*C*_*μ*_*C*_ after digestion by human gastric juice; the rate at which mature worms become fertilized females worms *α*_*M*_/2; and the rate at which mature worms and females worms die all contribute to the transmission of Guinea worm disease. Therefore immune mechanisms that kill infected copepods and worms within infected human host and also treatment intend to kill both mature worms and fertilized female worm population may help to reduce the transmission risk of GWD.

Therefore, both the epidemiological and immunological factors affect the transmission cycle of GWD in both humans and copepod population.

### 4.2. Local Stability of DFE

In this section we determine the local stability of DFE of the model system (1). We linearize equations of the model system (1) in order to obtain a Jacobian matrix. Then we evaluate the Jacobian matrix of the system at the disease-free equilibrium (DFE),

(29) $$\begin{array}{c} E_{0}=\left(\;\frac{\Lambda_{H}}{\mu_{H}},0,0,0,0,\frac{G_{0}}{\mu_{J}},0,0,\frac{\Lambda_{E}}{\mu_{E}},0\;\right). \end{array}$$

The Jacobian matrix of the model system (1) evaluated at the disease-free equilibrium state (DFE) is given by

(30) $$\begin{array}{c} J\left(\;E_{0}\;\right)=\left(\begin{array}{cccccccccc} -\mu_{H} & \alpha_{H} & 0 & 0 & 0 & 0 & 0 & 0 & 0 & -A_{0}\\ 0 & -q_{0} & 0 & 0 & 0 & 0 & 0 & 0 & 0 & A_{0}\\ 0 & 0 & -q_{1} & 0 & 0 & 0 & 0 & 0 & 0 & A_{1}\\ 0 & 0 & \frac{N_{C}\mu_{C}G_{0}}{\mu_{J}} & -q_{2} & 0 & 0 & 0 & 0 & 0 & 0\\ 0 & 0 & 0 & \frac{\alpha_{M}}{2} & -q_{3} & 0 & 0 & 0 & 0 & 0\\ 0 & 0 & \alpha_{J}\frac{G_{0}}{\mu_{J}} & 0 & 0 & -\mu_{J} & 0 & 0 & 0 & 0\\ 0 & 0 & 0 & 0 & \alpha_{F} & 0 & -q_{4} & 0 & 0 & 0\\ 0 & 0 & 0 & 0 & 0 & 0 & N_{W}\alpha_{W} & -\mu_{L} & 0 & 0\\ 0 & 0 & 0 & 0 & 0 & 0 & 0 & -\frac{\beta_{E}\Lambda_{E}}{\mu_{E}L_{0}} & -\mu_{E} & 0\\ 0 & 0 & 0 & 0 & 0 & 0 & 0 & \frac{\beta_{E}\Lambda_{E}}{\mu_{E}L_{0}} & 0 & -q_{5} \end{array}\right), \end{array}$$

where

(31) q0=μH+δH+αH,q1=μCG0+μJαCμJ,q2=μM+αM,q3=μF+αF,q4=μW+αW,q5=μE+αE,A0=βHΛHP0μH,A1=βHΛH−μHμHP0.

We consider stability of DFE by calculating the eigenvalues (*λ*_*s*_) of the Jacobian matrix given by (30). The characteristic equation for the eigenvalues is given by

(32) λ0λ6+π1λ5+π2λ4+π3λ3+π4λ2+π5λ+π6=0,

where

(33) $$\begin{array}{c} \lambda_{0}=\left(\;-\mu_{H}-\lambda \;\right)\left(\;-\mu_{E}-\lambda \;\right)\left(\;-\mu_{J}-\lambda \;\right)\left(\;-q_{0}-\lambda \;\right). \end{array}$$

It is clear from (32) that there are four negative eigenvalues (−*μ*_*H*_, −*μ*_*E*_, −*μ*_*J*_, and −*q*_0_). Now in order to make conclusions about the stability of the DFE, we use the Routh-Hurwitz criteria to determine the sign of the remaining eigenvalues of the polynomial

(34) $$\begin{array}{c} \lambda^{6}+\pi_{1}\lambda^{5}+\pi_{2}\lambda^{4}+\pi_{3}\lambda^{3}+\pi_{4}\lambda^{2}+\pi_{5}\lambda +\pi_{6}=0, \end{array}$$

where

(35) π1=q1+q2+q3+q4+q5+μL,π2=q1q2+q3q4+q1+q2+q3+q4q5+μL+q5μL+q1+q2q3+q4,π3=q1q2q3+q4+q3q4q1+q2+q1+q2q3+q4q5+μL+q1q2q5+μL+q3q4q5+μL+q5μLq1+q2+q3+q4,π4=q1q2q3q4+q3q4q1+q2q5+μL+q1q2q3+q4q5+μL+q1+q2q3+q4q1μL+q5q4q3μL+q1q2q5μL,π5=q1q2q3q4q5+μL+q3q4q1+q2q5μL+q1q2q3+q4q5μL,π6=q1q2q3q4q5μL1−R02.

Using the Routh-Hurwitz stability criterion, the equilibrium state associated with the model system (1) is stable if and only if the determinants of all the Hurwitz matrices associated with the characteristic equation (34) are positive; that is,

(36) $$\begin{array}{c} \mathrm{Det}\left(\;H_{j}\;\right)>0;\;j=1,2,\ldots ,6, \end{array}$$

where

(37) H1=π1;H2=π11π3π2;H3=π110π3π2π1π5π4π3;H4=π1100π3π2π11π5π4π3π20π6π5π4;H5=π11000π3π2π110π5π4π3π2π10π6π5π4π3000π6π5;H6=π110000π3π2π1100π5π4π3π2π110π6π5π4π3π2000π6π5π400000π6.

The Routh-Hurwitz criterion applied to (37) requires that the following conditions (H1)–(H6) be satisfied, in order to guarantee the local stability of the disease-free equilibrium point of the model system (1).

(H1) *π*_1_ > 0.
(H2) *π*_1_*π*_2_ − *π*_3_ > 0.
(H3) *π*_1_(*π*_2_*π*_3_ + *π*_5_) > *π*_1_*π*_4_ + *π*_3_^2^.
(H4) *π*_1_[*π*_2_(*π*_3_(*π*_4_ + *π*_5_) + *π*_1_*π*_6_)+(*π*_1_ + *π*_4_)] > *π*_1_[*π*_2_^2^*π*_5_ + *π*_3_*π*_6_ + *π*_1_*π*_4_^2^] + *π*_3_^2^*π*_4_ + *π*_5_^2^.
(H5) *π*_6_[*π*_1_(2*π*_2_*π*_5_ + *π*_3_(*π*_1_*π*_4_ − 3*π*_5_ − *π*_3_)) + *π*_3_^3^*π*_6_] + *π*_5_[*π*_5_(2*π*_1_*π*_4_ + *π*_2_*π*_3_ − *π*_1_*π*_2_(*π*_2_ + 1) + *π*_4_(*π*_1_*π*_2_*π*_3_ − *π*_1_^2^*π*_4_ − *π*_3_^2^))] > 0.
(H6) *π*_6_^2^[*π*_1_(2*π*_2_*π*_5_ + *π*_3_(*π*_1_*π*_4_ − 3*π*_5_ − *π*_3_)) + *π*_3_^3^*π*_6_] + *π*_5_*π*_6_[*π*_5_(2*π*_1_*π*_4_ + *π*_2_*π*_3_ − *π*_1_*π*_2_(*π*_2_ + 1) + *π*_4_(*π*_1_*π*_2_*π*_3_ − *π*_1_^2^*π*_4_ − *π*_3_^2^))] > 0.

From (37) we note that all the coefficients *π*_1_, *π*_2_, *π*_3_, *π*_4_, *π*_5_, and *π*_6_ of the polynomial *P*(*λ*) are greater than zero whenever *R*_0_^2^ < 1. And we also noted that the conditions above are satisfied if and only if *R*_0_^2^ < 1. Hence all the roots of the polynomial *P*(*λ*) either are negative or have negative real parts. The results are summarized in the following theorem.

**Theorem 1 .**

The disease-free equilibrium point of the model system (1) is locally asymptotically stable whenever *R*_0_ < 1.

### 4.3. Global Stability of DFE

To determine the global stability of DFE of the model system (1), we use Theorem 2 in [21] to establish that the disease-free equilibrium is globally asymptotically stable whenever *R*_0_ < 1 and unstable when *R*_0_ > 1. In this section, we list two conditions that if met, also guarantee the global asymptotic stability of the disease-free state. We write the model system (1) in the form

(38) dXdt=FX,Z,dYdt=GX,Z,

where

- *X* = (*S*_*H*_, *S*_*E*_, *G*_*J*_) represents all uninfected components.
- *Z* = (*I*_*H*_, *I*_*C*_, *W*_*M*_, *W*_*F*_, *E*_*W*_, *L*_*W*_, *I*_*E*_) represents all compartments of infected and infectious components.

We let

(39) $$\begin{array}{c} U_{0}=\left(\;X^{*},0\;\right)=\left(\;\frac{\Lambda_{H}}{\mu_{H}},0,0,\frac{\Lambda_{C}}{\mu_{C}},0,0,0\;\right) \end{array}$$

denote the disease-free equilibrium (DFE) of the system. To guarantee global asymptotic stability of the disease-free equilibrium, conditions (H1) and (H2) below must be met [20].

(H1) *dX*/*dt* = *F*(*X*, 0) is globally asymptotically stable,
(H2) $G(X,Z)=AZ-\hat{G}(X,Z)$ and $\hat{G}(X,Z)\geq 0$ for (*X*, *Z*) ∈ *ℝ*_+_^10^, where *A* = *D*_*Z*_*G*(*X*^*∗*^, 0) is an *M*-matrix and *ℝ*_+_^10^ is the region where the model makes biological sense.

In our case

(40) $$\begin{array}{c} F\left(\;X,0\;\right)=\left[\begin{array}{c} \Lambda_{H}-\mu_{H}S_{H}\\ \Lambda_{E}-\mu_{E}S_{E}\\ G_{0}-\mu_{J}G_{J} \end{array}\right]. \end{array}$$

Matrix *A* is given by

(41) $$\begin{array}{c} A=\left[\begin{array}{ccccccc} -a_{0} & 0 & 0 & 0 & 0 & 0 & \frac{\beta_{H}\Lambda_{H}}{P_{0}\mu_{H}}\\ 0 & -a_{1} & 0 & 0 & 0 & 0 & \frac{\beta_{H}\left(\Lambda_{H}-\mu_{H}\right)}{\mu_{H}P_{0}}\\ 0 & N_{C}\mu_{C}\frac{G_{0}}{\mu_{J}} & -a_{2} & 0 & 0 & 0 & 0\\ 0 & 0 & \frac{\alpha_{M}}{2} & -a_{3} & 0 & 0 & 0\\ 0 & 0 & 0 & \alpha_{F} & -a_{4} & 0 & 0\\ 0 & 0 & 0 & 0 & N_{W}\alpha_{W} & -\mu_{L} & 0\\ 0 & 0 & 0 & 0 & 0 & \frac{\beta_{E}\Lambda_{E}}{L_{0}\mu_{E}} & -a_{5} \end{array}\right], \end{array}$$

where

(42) a0=μH+δH+αH,a1=1μJμC+αCμJ,a2=μM+αM,a3=μF+αF,a4=μW+αW,a5=μE+αE,G^X,Z=ΛHμHP0−SHP0+ϵIEβHIEΛH−μHμHP0−SH−1P0+ϵIEβHIE+ICμCGJ−G0μJ+αC1−μJ0000ΛEμEL0−SEL0+ϵLWβELW.

Assume that *G*_*J*_ = *G*_0_/*μ*_*J*_ and *μ*_*J*_ ∈ [0,1]. It is clear that $\hat{G}(X,Z)\geq 0$ for all (*X*, *Z*) ∈ *ℝ*_+_^10^, since Λ_*H*_/*μ*_*H*_*P*_0_ ≥ *S*_*H*_/(*P*_0_ + *ϵI*_*E*_), Λ_*E*_/*μ*_*E*_*L*_0_ ≥ *S*_*E*_/(*L*_0_ + *ϵL*_*W*_), and (Λ_*H*_ − *μ*_*H*_)/*μ*_*H*_*P*_0_ ≥ (*S*_*H*_ − 1)/(*P*_0_ + *ϵI*_*C*_) provided that Λ_*H*_ > *μ*_*H*_. It is also clear that *A* is an *M*-matrix, since the off diagonal elements of *A* are nonnegative. We state a theorem which summarizes the above result.

**Theorem 2 .**

The disease-free equilibrium of model system (1) is globally asymptotically stable if *R*_0_ ≤ 1 and the assumptions (H1) and (H2) are satisfied.

## 5. The Endemic Equilibrium State and Its Stability

At the endemic equilibrium humans are infected by copepods that have been infected by first-stage larvae (*L*_*W*_). The endemic equilibrium point of the model system (1) given by

(43) $$\begin{array}{c} {\hat{E}}_{1}=\left(\;S_{H}^{*},I_{H}^{*},I_{C}^{*},W_{M}^{*},W_{F}^{*},G_{J}^{*},E_{W}^{*},L_{W}^{*},S_{E}^{*},I_{E}^{*}\;\right) \end{array}$$

satisfies

(44) 0=ΛH−λH∗SH∗−μHSH∗+αHIH∗,0=λH∗SH∗−μH+δH+αHIH∗,0=λh∗Sh∗−μCGJ∗IC∗−αCIC∗,0=NCμCGJ∗IC∗−αM+μMWM∗,0=αM2WM∗−μF+αFWF∗,0=G0+αJGJ∗IC∗−μJGJ∗,0=αFWF∗Ih∗−μW+αWEW∗,0=NWαWEW∗−μLLW∗,0=ΛE−λE∗SE∗−μESE∗,0=λE∗SE∗−μE+δEIE∗,

for all *S*_*H*_^*∗*^, *I*_*H*_^*∗*^, *I*_*C*_^*∗*^, *W*_*M*_^*∗*^, *W*_*F*_^*∗*^, *G*_*J*_^*∗*^, *E*_*W*_^*∗*^, *L*_*W*_^*∗*^, *S*_*E*_^*∗*^, *I*_*E*_^*∗*^ > 0. We therefore obtain the following endemic values. The endemic value of susceptible humans is given by

(45) $$\begin{array}{c} S_{H}^{*}=\frac{\Lambda_{H}+\alpha_{H}I_{H}^{*}}{\left(\lambda_{H}^{*}+\mu_{H}\right)}. \end{array}$$

From (45) we note that the susceptible human population at endemic equilibrium is proportional to the average time of stay in the susceptible class and the rate at which new susceptible individuals are entering the susceptible class either through birth or through infected individuals who recover from the disease. Individuals leave the susceptible class through either infection or death. The endemic value of infected humans is given by

(46) $$\begin{array}{c} I_{H}^{*}=\frac{\lambda_{H}^{*}S_{H}^{*}}{\left(\mu_{H}+\delta_{H}+\alpha_{H}\right)}. \end{array}$$

We note from (46) that the population of infected humans at the endemic equilibrium point is proportional to the average time of stay in the infected class, the rate at which susceptible individuals become infected, and the density of susceptible individuals. The endemic value of infected copepods population within a single infected human at the equilibrium point is given by

(47) $$\begin{array}{c} I_{C}^{*}=\frac{\lambda_{H}^{*}\left(S_{H}^{*}-1\right)}{\left(I_{H}^{*}+1\right)\left(\mu_{C}G_{J}^{*}+\alpha_{C}\right)}, \end{array}$$

where *S*_*H*_^*∗*^ > 1. From (47) we note that the average infected copepod population within a single infected human is proportional to the average life-span of infected copepods within a single infected human host and the rate of infection of a single susceptible individual to become infected. We also note that this expression provides a link between the dynamics of the infected copepods within-host and human population dynamics. The endemic value of mature worm population within a single infected human is given by

(48) $$\begin{array}{c} W_{M}^{*}=\frac{N_{C}\mu_{C}G_{J}^{*}I_{C}^{*}}{\left(\alpha_{M}+\mu_{M}\right)}. \end{array}$$

We note from (48) that the population of mature worms within a single infected human at endemic equilibrium point is proportional to the average life-span of mature worms and the rate at which mature worms are released after infected copepods within human host have been killed by human gastric juice. The endemic value of fertilized female worm population within a single infected human is given by

(49) $$\begin{array}{c} W_{F}^{*}=\frac{1}{2}\frac{\alpha_{M}W_{M}^{*}}{\left(\alpha_{F}+\mu_{F}\right)}. \end{array}$$

The average population of fertilized female worms within an infected human at endemic equilibrium point is equal to the average life-span of female worms and the rate at which mature worms become fertilized female worms. The endemic value of a single human gastric juice is given by

(50) $$\begin{array}{c} G_{J}^{*}=\frac{G_{0}}{\left(\mu_{J}-\alpha_{J}I_{C}^{*}\right)}, \end{array}$$

where *μ*_*J*_ > *α*_*J*_*I*_*C*_^*∗*^. The endemic value of Guinea worm eggs population in the physical water environment is given by

(51) $$\begin{array}{c} E_{W}^{*}=\frac{\alpha_{F}W_{F}^{*}\left(I_{H}^{*}+1\right)}{\left(\alpha_{W}+\mu_{W}\right)}. \end{array}$$

We note from (51) that the worm egg population at equilibrium point is proportional to the average life-span of eggs, the rate at which each infected human host excretes Guinea worm eggs, and the total number of infected humans. The endemic value of Guinea worm larva population in the physical water environment is given by

(52) $$\begin{array}{c} L_{W}^{*}=\frac{N_{W}\alpha_{W}E_{W}^{*}}{\mu_{L}}. \end{array}$$

We note from (52) that the larvae population at equilibrium point is proportional to the rate at which Guinea worm eggs hatch, the number of larvae generated by each egg, and the average life-span of larvae. The value of susceptible copepod population at equilibrium point is given by

(53) $$\begin{array}{c} S_{E}^{*}=\frac{\Lambda_{E}}{\left(\lambda_{E}^{*}+\mu_{E}\right)}. \end{array}$$

From (53) we note that susceptible copepod population at endemic equilibrium is proportional to the average time of stay in susceptible copepod class and the rate at which new susceptible copepods are entering the susceptible copepod class through birth. The endemic value of infected copepod population is given by

(54) $$\begin{array}{c} I_{E}^{*}=\frac{\lambda_{E}^{*}S_{E}^{*}}{\left(\delta_{E}+\mu_{E}\right)}=\frac{\lambda_{E}^{*}\Lambda_{E}}{\left(\lambda_{E}^{*}+\mu_{E}\right)\left(\delta_{E}+\mu_{E}\right)}. \end{array}$$

We note from (54) that infected copepod population at the endemic equilibrium point is proportional to the average time of stay in the infected copepod class, the rate at which susceptible copepods become infected, and the density of susceptible copepods. We also make the endemic equilibrium of the model system (1) given by expressions (45)–(54) depend on both within-host and between-host disease parameters.

### 5.1. Existence of the Endemic Equilibrium State

In this section we present some results concerning the existence of an endemic equilibrium solution for the model system (1). To determine the existence and uniqueness of the endemic equilibrium point (EEP) of the model system (1), we can easily express *S*_*H*_^*∗*^, *I*_*H*_^*∗*^, *I*_*C*_^*∗*^, *W*_*M*_^*∗*^, *W*_*F*_^*∗*^, *E*_*W*_^*∗*^, and *L*_*W*_^*∗*^ in terms of *I*_*E*_^*∗*^ in the form

(55) SH∗IE∗=ΛHa1+a2IE∗+αHa0IE∗P0+ϵIE∗a1+a2IH∗βHIE∗+μHP0+ϵIE∗,IH∗IE∗=a0IE∗a1+a2IE∗,IC∗IE∗=IE∗βHΛH−μHZEa+ZEbβHIE∗P0+ϵIE∗μCHGJ∗+αCIH∗+1ZEc,WM∗IE∗=NCμCGJ∗IE∗βHΛH−μHZEa+ZEbβHIE∗αM+μMP0+ϵIE∗μCGJ∗+αCIH∗+1ZEc,WF∗IE∗=12αMNCμCGJ∗IE∗βHΛH−μHZEa+ZEbβHIE∗αF+μFαM+μMP0+ϵIE∗μCHGJ∗+αCIH∗+1ZEc,EW∗IE∗=αFαMNCμCGJ∗IE∗βHΛH−μHZEa+ZEbβHIE∗2αW+μWαF+μFαM+μMP0+ϵIE∗μCHGJ∗+αCZEc,LW∗IE∗=QEGJ∗μCGJ∗+αC·βHΛH−μHIE∗ZEa+ZEbβHIE∗2P0+ϵIE∗ZEc,

where

(56) ZEa=a1+a2IE∗P0+ϵIE∗,ZEb=ΛHαHa0P0+ϵIE∗−a1+a2IE∗2βH,ZEc=a1+a2IE∗βHIE∗+μHP0+ϵIE∗,QE=12·NCμCμL·NWαWμW+αW·αFμF+αF·αMμM+αM,a0=βHΛH,a1=μHP0μH+δH+αH,a2=βHμH+δHμHϵμH+δH+αH.

Substituting the expression *λ*_*E*_ = *β*_*E*_*L*_*W*_/(*L*_0_ + *ϵL*_*W*_) and *L*_*W*_^*∗*^ = *Q*_*E*_*G*_*J*_^*∗*^/(*μ*_*C*_*G*_*J*_^*∗*^ + *α*_*C*_) · ((*β*_*H*_(Λ_*H*_ − *μ*_*H*_)*I*_*E*_^*∗*^*Z*_*E*_^(*a*)^ + *Z*_*E*_^(*b*)^*β*_*H*_*I*_*E*_^*∗*2^)/(*P*_0_ + *ϵI*_*E*_^*∗*^)*Z*_*E*_^(*c*)^) into (25) we get

(57) $$\begin{array}{c} I_{E}^{*}h\left(\;I_{E}^{*}\;\right)=I_{E}^{*}\left[\;\gamma_{3}I_{E}^{*3}+\gamma_{2}I_{E}^{*2}+\gamma_{1}I_{E}^{*}+\gamma_{0}\;\right]=0, \end{array}$$

where

(58) γ3=GJ∗μCG0+μJαCβE+ϵμEL0μEP0μHϵβEΛEG0μCGJ∗+αCR02a2βH−μH+a2βH+μHϵμEL0−βHΛHαHa0>0,γ1=GJ∗μCG0+μJαCβE+ϵμEL0μEμE+δHP02μHa1+ΛHαHa0βEΛEG0μCGJ∗+αCR02+a11−βHμE+δEμCG0+μJαCG0R02+P0μEL0a2βH+μHϵ−L0μEP0μHμCG0+μJαCGJ∗G0μCGJ∗+αCa1ϵ+a2P0+ΛHG0ϵΛH−μH,γ2=BβHΛH−μHa1ϵ+a2P0+a2μEL0βH+ϵμEΛH−μH+AβHβH−ΛH−μHϵ,γ0=μEL0μHa1P01−GJ∗μCG0+μJδCμCGJ∗+αCG0R02−GJ∗μCG0+μJαCL0μEP02μHβHΛH−μHG0μCGJ∗+αCμHμH+δH+αH−ΛH2αHR02,A=QEGJ∗βEΛEμCGJ∗+αCμE+δE,B=QEGJ∗βE+ϵμEμCGJ∗+αC.

We can easily note that (57) gives *I*_*E*_^*∗*^ = 0, which corresponds to the disease-free equilibrium and

(59) $$\begin{array}{c} h\left(\;I_{E}^{*}\;\right)=\gamma_{3}I_{E}^{*3}+\gamma_{2}I_{E}^{*2}+\gamma_{1}I_{E}^{*}+\gamma_{0}=0, \end{array}$$

which corresponds to the existence of endemic equilibria. Solving for *I*_*E*_^*∗*^ in *h*(*I*_*E*_^*∗*^) = 0, the roots of *h*(*I*_*E*_^*∗*^) = 0 are determined by using Descartes's rule of sign. The various possibilities are tabulated in Table 2.

We summarize the results in Table 2 in the following Theorem 3.

**Theorem 3 .**

The model system (1)

1. has a unique endemic equilibrium whenever Cases  1, 2, 3, 4, 5, 6, 7, and 8 are satisfied and if *R*_0_ > 1,
2. could have more than one endemic equilibrium if Case  8 is satisfied and *R*_0_ > 1,
3. could have two endemic equilibria if Cases  3, 5, and 7 are satisfied.

We now employ the center manifold theory [22] to establish the local asymptotic stability of the endemic equilibrium of model system (1).

### 5.2. Local Stability of the Endemic Equilibrium

We determine the local asymptotic stability of the endemic steady state of the model system (1) by using the center manifold theory described in [22]. In our case, we use center manifold theory by making the following change of variables. Let *S*_*H*_ = *x*_1_, *I*_*H*_ = *x*_2_, *I*_*C*_ = *x*_3_, *W*_*M*_ = *x*_4_, *W*_*F*_ = *x*_5_, *G*_*J*_ = *x*_6_, *E*_*W*_ = *x*_7_, *E*_*W*_ = *x*_8_, *S*_*E*_ = *x*_9_, and *I*_*E*_ = *x*_10_. We also use the vector notation **x** = (*x*_1_, *x*_2_, *x*_3_, *x*_4_, *x*_5_, *x*_6_, *x*_7_, *x*_8_, *x*_9_, *x*_10_)^*T*^ so that the model system (1) can be written in the form

(60) $$\begin{array}{c} \frac{dx}{dt}=f\left(\;x,\beta^{*}\;\right), \end{array}$$

where

(61) $$\begin{array}{c} f=\left(\;f_{1},f_{2},f_{3},f_{4},f_{5},f_{6},f_{7},f_{8},f_{9},f_{10}\;\right). \end{array}$$

Therefore, model system (1) can be rewritten as

(62) x˙1=ΛH−λHx1−μHx1+αHx2,x˙2=λHx1−μH+δH+αHx2,x˙3=λHx1−1x2+1−μCx6+αCx3,x˙4=NCμCx6x3−αM+μMx4,x˙5=αM2x4−μF+αFx5,x˙6=G0+αJx6x3−μJx6,x˙7=αFx5x2+1−μW+αWx7,x˙8=NWαWx7−μLx8,x˙9=ΛE−λEx9−μEx9,x˙10=λEx9−μE+δEx10,

where

(63) λH=β∗x10P0+ϵx10,λE=kβ∗x8L0+ϵx8.

The method involves evaluating the Jacobian matrix of system (62) at the disease-free equilibrium *E*^0^ denoted by *J*(*E*^0^). The Jacobian matrix associated with the system of (62) evaluated at the disease-free equilibrium (*E*_0_) is given by

(64) $$\begin{array}{c} J\left(\;E_{0}\;\right)=\left(\begin{array}{cccccccccc} -\mu_{H} & \alpha_{H} & 0 & 0 & 0 & 0 & 0 & 0 & 0 & -\frac{\beta_{H}\Lambda_{H}}{\mu_{H}P_{0}}\\ 0 & b_{0} & 0 & 0 & 0 & 0 & 0 & 0 & 0 & \frac{\beta_{H}\Lambda_{H}}{P_{0}\mu_{H}}\\ 0 & 0 & b_{1} & 0 & 0 & 0 & 0 & 0 & 0 & \frac{\beta_{H}\left(\Lambda_{H}-\mu_{H}\right)}{\mu_{H}P_{0}}\\ 0 & 0 & \frac{N_{C}\mu_{C}G_{0}}{\mu_{J}} & b_{2} & 0 & 0 & 0 & 0 & 0 & 0\\ 0 & 0 & 0 & \frac{\alpha_{M}}{2} & b_{3} & 0 & 0 & 0 & 0 & 0\\ 0 & 0 & \alpha_{J}\frac{G_{0}}{\mu_{J}} & 0 & 0 & -\mu_{J} & 0 & 0 & 0 & 0\\ 0 & 0 & 0 & 0 & \alpha_{F} & 0 & b_{4} & 0 & 0 & 0\\ 0 & 0 & 0 & 0 & 0 & 0 & N_{W}\alpha_{W} & -\mu_{L} & 0 & 0\\ 0 & 0 & 0 & 0 & 0 & 0 & 0 & -\frac{\beta_{E}\Lambda_{E}}{\mu_{E}L_{0}} & -\mu_{E} & 0\\ 0 & 0 & 0 & 0 & 0 & 0 & 0 & \frac{\beta_{E}\Lambda_{E}}{\mu_{E}L_{0}} & 0 & b_{5} \end{array}\right), \end{array}$$

where

(65) b0=−μH+δH+αH,b1=−μCG0+μJαCμJ,b2=−μM+αM,b3=−μF+αF,b4=−μW+αW,b5=−μE+αE.

By using the similar approach from Section 4.1, the basic reproductive number of model system (62) is

(66) $$\begin{array}{c} R_{0}=\sqrt{\frac{1}{2}\cdot \frac{\alpha_{M}}{\alpha_{M}+\mu_{M}}\cdot \frac{\alpha_{F}}{\alpha_{F}+\mu_{F}}\cdot \frac{N_{C}\mu_{C}G_{0}}{\mu_{C}G_{0}+\mu_{J}\alpha_{C}}\cdot \frac{\beta_{H}\left(\Lambda_{H}-\mu_{H}\right)}{P_{0}\mu_{H}}\cdot \frac{N_{W}\alpha_{W}}{\left(\alpha_{W}+\mu_{W}\right)\mu_{L}}\frac{\beta_{E}\Lambda_{E}}{\mu_{E}\left(\mu_{E}+\delta_{E}\right)L_{0}}}. \end{array}$$

Now let us consider *β*_*E*_ = *kβ*_*H*_, regardless of whether *k* ∈ (0,1) or *k* ≥ 1, and let *β*_*H*_ = *β*^*∗*^. Taking *β*^*∗*^ as the bifurcation parameter and if we consider *R*_0_ = 1 and solve for *β*^*∗*^ in (66), we obtain

(67) $$\begin{array}{c} \beta^{*}=\sqrt{\frac{2L_{0}\left(\mu_{E}+\delta_{E}\right)\mu_{E}\left(\mu_{W}+\alpha_{W}\right)\mu_{L}\left(\mu_{M}+\alpha_{M}\right)\left(\mu_{F}+\alpha_{F}\right)\left(\mu_{C}G_{0}+\alpha_{C}\mu_{J}\right)P_{0}\mu_{H}}{k\alpha_{F}\alpha_{M}N_{C}\mu_{C}G_{0}N_{W}\alpha_{W}\left(\Lambda_{H}-\mu_{H}\right)\Lambda_{E}}}. \end{array}$$

Note that the linearized system of the transformed equations (62) with bifurcation point *β*^*∗*^ has a simple zero eigenvalue. Hence, the center manifold theory [22] can be used to analyse the dynamics of (62) near *β*_*H*_ = *β*^*∗*^.

In particular, Theorem  4.1 in Castillo-Chavez and Song [23], reproduced below as Theorem 4 for convenience, will be used to show the local asymptotic stability of the endemic equilibrium point of (62) (which is the same as the endemic equilibrium point of the original system (1), for *β*_*H*_ = *β*^*∗*^).

**Theorem 4 .**

Consider the following general system of ordinary differential equations with parameter *ϕ*:

(68) dxdt=fx,ϕ,f:Rn×R⟶R,  f:C2R2×R,

where 0 is an equilibrium of the system, that is, *f*(0, *ϕ*) = 0 for all *ϕ*, and assume that

(A1) *A* = *D*_*x*_*f*(0,0) = ((∂*f*_*i*_/∂*x*_*j*_)(0,0)) is a linearization matrix of the model system (68) around the equilibrium 0 with *ϕ* evaluated at 0. Zero is a simple eigenvalue of *A*, and other eigenvalues of *A* have negative real parts,
(A2) matrix *A* has a right eigenvector *u* and a left eigenvector *v* corresponding to the zero eigenvalue.

Let *f*_*k*_ be the *k*th component of *f* and

(69) a=∑k,i,j=1nukvivj∂2fk∂xi∂xj0,0,b=∑k,i=1nukvi∂2fk∂xi∂ϕ0,0.

The local dynamics of (68) around 0 are totally governed by *a* and *b* and are summarized as follows.

- *a* > 0 and *b* > 0. When *ϕ* < 0 with |*ϕ*| ≪ 1, 0 is locally asymptotically stable, and there exists a positive unstable equilibrium; when 0 < *ϕ* ≪ 1, 0 is unstable and there exists a negative and locally asymptotically stable equilibrium.
- *a* < 0 and *b* < 0. When *ϕ* < 0 with |*ϕ* | ≪ 1, 0 is unstable; when 0 < *ϕ* ≪ 1, 0 is locally asymptotically stable, and there exists a positive unstable equilibrium.
- *a* > 0 and *b* < 0. When *ϕ* < 0 with |*ϕ* | ≪ 1, 0 is unstable, and there exists a locally asymptotically stable negative equilibrium; when 0 < *ϕ* ≪ 1, 0 is stable and a positive unstable equilibrium appears.
- *a* < 0 and *b* > 0. When *ϕ* changes from negative to positive, 0 changes its stability from stable to unstable. Correspondingly a negative unstable equilibrium becomes positive and locally asymptotically stable.

In order to apply Theorem 4, the following computations are necessary (it should be noted that we are using *β*^*∗*^ as the bifurcation parameter, in place of *ϕ* in Theorem 4).

*Eigenvectors of J*_*β*^*∗*^_. For the case when *R*_0_ = 1, it can be shown that the Jacobian matrix of (62) at *β*_*H*_ = *β*^*∗*^ (denoted by *J*_*β*^*∗*^_) has a right eigenvector associated with the zero eigenvalue given by

(70) $$\begin{array}{c} u={\left[\;u_{1},u_{2},u_{3},u_{4},u_{5},u_{6},u_{7},u_{8},u_{9},u_{10},u_{11},u_{12}\;\right]}^{T}, \end{array}$$

where

(71) u1=β∗ΛHμH2P0αHμH+δH+αH−1,u2=β∗ΛHμH+δH+αHP0μH,u3=β∗ΛH−μHP0μHμJμCG0+μJαC,u4=NCαCG0μCG0+μJαC·β∗ΛH−μHP0μHμM+αM,u5=αM2μM+αMμF+αFNCμCG0μCG0+μJαC·β∗ΛH−μHP0μH,u6=αJG0μJμCG0+μJαC·β∗ΛH−μHP0μH,u7=αMαF2μM+αMμF+αFNCμCG0μCG0+μJαC·β∗ΛH−μHP0μH1μW+αW,u8=αMαF2μM+αMμF+αFNCμCG0μCG0+μJαC·β∗ΛH−μHP0μHNWαWμLμW+αW,u9=−αMαF2μM+αMμF+αFNCμCG0μCG0+μJαC·β∗2ΛH−μHP0μHNWαWμLμW+αW·kΛEL0μE2.u10=1.

In addition, the left eigenvector of the Jacobian matrix in (64) associated with the zero eigenvalue at *β*_*H*_ = *β*^*∗*^ is given by

(72) $$\begin{array}{c} v={\left[\;v_{1},v_{2},v_{3},v_{4},v_{5},v_{6},v_{7},v_{8},v_{9},v_{10},v_{11},v_{12}\;\right]}^{T}, \end{array}$$

where

(73) v1=0,v2=0v3=1,v4=β∗2ΛH−μHμHP0·αFαM2μF+αFμM+αM·NWαWμLμW+αW·kΛEμEμE+δEL0,v5=β∗2ΛH−μHμHP0·αFμF+αF·NWαWμLμW+αW·kΛEμEμE+δEL0,v6=0,v7=β∗2ΛH−μHμHP0·NWαWμLμW+αW·kΛEμEμE+δEL0,v8=β∗2ΛH−μHμHP0·1μL·kΛEμEμE+δEL0,v9=0,v10=β∗ΛH−μHμE+δEμHP0.

*Computation of Bifurcation Parameters a and b*. We evaluate the nonzero second-order mixed derivatives of** f** with respect to the variables and *β*^*∗*^ in order to determine the signs of *a* and *b*. The sign of *a* is associated with the following nonvanishing partial derivatives of **f**:

(74) ∂2f1∂x102=2ϵβ∗ΛHP02μH,∂2f2∂x102=−2ϵβ∗ΛHP02μH,∂2f3∂x102=−2ϵβ∗ΛH−μHP02μH,∂2f9∂x82=2ϵkβ∗ΛEL02μE,∂2f10∂x82=−2ϵkβ∗ΛEL02μE.

The sign of *b* is associated with the following nonvanishing partial derivatives of** f**:

(75) ∂2f1∂x10∂β∗=−ΛHμHP0,∂2f2∂x10∂β∗=ΛHμHP0,∂2f3∂x10∂β∗=ΛH−μHμHP0,∂2f9∂x8∂β∗=−kΛEμEL0,∂2f10∂x8∂β∗=kΛEμEL0.

Substituting expressions (71), (73), and (74) into (69), we get

(76) a=u1v102∂2f1∂x102+u2v102∂2f2∂x102+u3v102∂2f3∂x102+u9v82∂2f9∂x82+u10v82∂2f10∂x82=u1v1022ϵβ∗ΛHP02μH+u2v102−2ϵβ∗ΛHP02μH+u3v102−2ϵβ∗ΛH−μHP02μH+u9v822ϵkβ∗ΛEL02μE+u10v82−2ϵkβ∗ΛEL02μE=2ϵβ∗ΛHP02μH·v102u1−u2−u3v1022ϵβ∗ΛH−μHP02μH+2ϵkβ∗ΛEL02μE·v82u9−u10<0

since (*u*_1_ − *u*_2_) < 0, (*u*_9_ − *u*_10_) < 0, *u*_3_ > 0, and *v*_10_ > 0.

Similarly, substituting expressions (71) and (73) and (75) into (69), we get

(77) b=u1v10∂2f1∂x10∂β∗+u2v10∂2f2∂x10∂β∗+u3v10∂2f3∂x8∂β∗+u9v8∂2f9∂x10∂β∗+u10v8∂2f10∂x8∂β∗=v10ΛHP0μH·u2−ΛHP0μH·u1+ΛH−μHP0μH·u3+kΛEL0μE·v8u10−u9=ΛHP0μHv10u2−u1+ΛH−μHP0μHv10u3+kΛEL0μEv8u10−u9>0,

since (*u*_2_ − *u*_1_) > 0, (*u*_10_ − *u*_9_) > 0, *u*_3_ > 0, and *v*_10_ > 0.

Thus, *a* < 0 and *b* > 0. Using Theorem 4, item (iv), we have established the following result which only holds for *R*_0_ > 1 but close to 1.

**Theorem 5 .**

The endemic equilibrium guaranteed by Theorem 3 is locally asymptotically stable for *R*_0_ > 1 near 1.

## 6. Sensitivity Analysis

In this section we carry out sensitivity analysis to evaluate the relative change in basic reproduction number (*R*_0_) when the within-host and between-host parameters as well as the environmental parameters of the model system (1) change. We used the normalized forward sensitivity index of the basic reproduction number, *R*_0_ of the model system (1) to each of the model parameters. The normalized forward sensitivity index of a variable to a parameter is typically defined as “the ratio of the relative change in the variable to the relative change in the parameter” [24]. In this case, if we let *R*_0_ be a differentiable function of the parameter *u*, then the normalized forward sensitivity index of *R*_0_ at *u* is defined as

(78) $$\begin{array}{c} \Upsilon_{u}^{R_{0}}=\frac{\partial R_{0}}{\partial u}\times \frac{u}{R_{0}}, \end{array}$$

where the quotient *u*/*R*_0_ is introduced to normalize the coefficient by removing the effect of units [25]. For example, the sensitivity index of *R*_0_ with respect to the human infection rate *β*_*H*_ is given by

(79) $$\begin{array}{c} \Upsilon_{\beta_{H}}^{R_{0}}=\frac{\partial R_{0}}{\partial \beta_{H}}\times \frac{\beta_{H}}{R_{0}}=0.5. \end{array}$$

It can be easily noted that the sensitivity index of *R*_0_ with respect to the parameter *β*_*H*_ does not depend on any of the parameter values. The indices of worm larvae death rate within a host and copepods death rate in the physical environment are, respectively, given by

(80) ΥμFR0=−12μFμF+αF=−0.5,ΥμER0=−122μE+δEμE+δE=−0.9991.

Using (78)–(80) similar expressions can be derived for the remaining parameters. The resulting sensitivity indices of *R*_0_ to the different model parameters are shown in Table 3. We see from (78)–(80) that the index of parameter *β*_*H*_ is positive and indexes of both parameters *μ*_*L*_ and *μ*_*E*_ are negative. The sign of the index value indicates whether the parameter increases the reproduction number or reduces the reproduction number. Therefore increasing human infection rate *β*_*H*_ reduces *R*_0_ and also increasing *μ*_*L*_ or *μ*_*E*_ reduces *R*_0_. Based on the results shown in Table 3, we observe that the reproduction number *R*_0_ is sensitive to the changes of both the within-host and between-host parameters as well as the environmental parameters (parameters which can be modified by environmental conditions which impact on survival and reproduction of the parasite and vector populations). More specifically, we deduce the following results for the between-host scale:

- The reproductive number is most sensitive to the changes of parameter *μ*_*E*_ and the natural death rate of copepods in the physical water environment. This implies that interventions focused on vector control have highest impact on GWD control. Since *Υ*_*μ*_*E*__^*R*_0_^ = −0.9991, increasing *μ*_*E*_ by 10% decreases the reproduction number by 9.991%. Therefore increasing the death rate of copepods by using chemical such as ABATE or temephos will eventually reduce the transmission of Guinea worm disease.
- The reproductive number also shows significant sensitivity to *β*_*H*_ and *β*_*E*_ since *Υ*_*β*_*H*__^*R*_0_^ = *Υ*_*β*_*E*__^*R*_0_^ = 0.5. This implies that reducing human infection rates *β*_*H*_ and *β*_*E*_ by 10% reduces *R*_0_ by 5% for each of these parameters. Therefore, health education to ensure that greater numbers of individuals and communities adopt behavioural practices such as voluntary reporting of GWD cases, prevention of GWD patients from entering drinking water bodies, regular use of water from safe water sources, and, in the absence of such water sources, filtering or boiling water before drinking aimed at preventing transmission of GWD would have high impact in complementing vector control in elimination of GWD.
- Similarly, *Υ*_*α*_*F*__^*R*_0_^ = 0.4639. This implies that reducing the rate at which eggs are excreted in the physical water environment, *α*_*F*_, by 10% reduces *R*_0_ by 4.639%. Therefore educating people about GWD (i.e., teaching people not to immerse their infected feet into the drinking water when the fertilized female worm is emerging out from their feet or to always filter contaminated water before drinking the water) will reduce the transmission of the disease.

Further, we also deduce the following results for the within-host scale:

- The development of a drug that would kill mature worms within human host would have significant benefits at within-host. However, the drug would have even higher impact if it would kill fertilized female worms.
- The development of interventions that would increase the supply rate of gastric juice would have no benefits in the control of GWD.

Therefore, the lack of drugs to treat GWD has delayed progress in eliminating GWD.

## 7. Numerical Analysis

The behaviour of model system (1) was investigated using numerical simulations using a Python program version V 2.6 on the Linux operation system (Ubuntu 14.04). The program uses a package odeint function in the scipy.integrate for solving a system of differential equations. The behaviour of the system model (1) was simulated in order to illustrate the analytical results we obtained in this paper. We used parameter values presented in Tables 4–6. Some of the parameter values used in the numerical simulations are from published literature while others were estimated as values of some parameters are generally not reported in literature. The initial conditions used for simulations are given by *S*_*H*_(0) = 2500, *I*_*H*_(0) = 10, *I*_*C*_(0) = 0, *G*_*J*_(0) = 1.50, *W*_*M*_(0) = 0, *W*_*F*_(0) = 0, *S*_*E*_(0) = 100000, *I*_*E*_(0) = 0, *E*_*W*_(0) = 0, and *L*_*W*_(0) = 50000.

**Figure 2:**
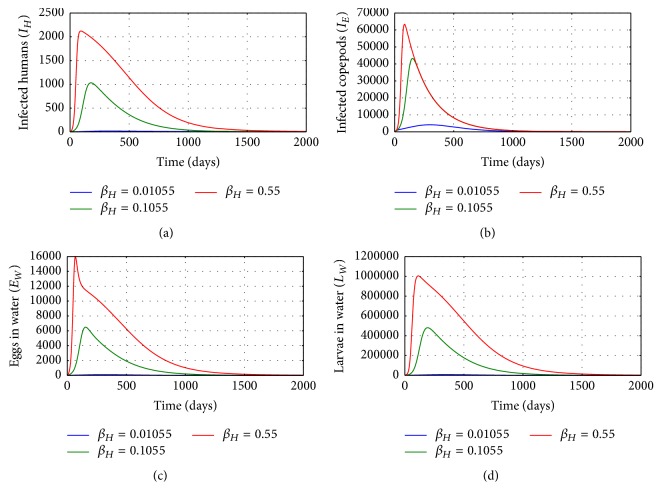
Graphs of numerical solutions of model system (1) showing the evolution with time of (a) population of infected humans (*I*_*H*_), (b) population of infected copepods (*I*_*E*_), (c) population of Guinea worm eggs in the physical water environment, and (d) population of Guinea worm larvae in the physical water environment, for different values of the infection rate of humans *β*_*H*_: *β*_*H*_ = 0.1055, *β*_*H*_ = 0.55, and *β*_*H*_ = 0.9.

Figure 2 illustrates the solution profile of the population of (a) infected humans, (b) infected copepods in the physical water environment, (c) worm eggs in the physical water environment, and (d) worm larvae in the physical water environment, for different values of the infection rate of humans *β*_*H*_: *β*_*H*_ = 0.1055, *β*_*H*_ = 0.55, and *β*_*H*_ = 0.9. The numerical results show that higher rates of infection at the human population level result in increased population of parasites (worm eggs and worm larvae) in the physical water environment and a noticeable increase in infected copepod population in the physical water environment. Therefore, human behavioural changes which reduce contact with contaminated water bodies through drinking contaminated water reduce transmission of the disease at both human and copepod population level.

**Figure 3:**
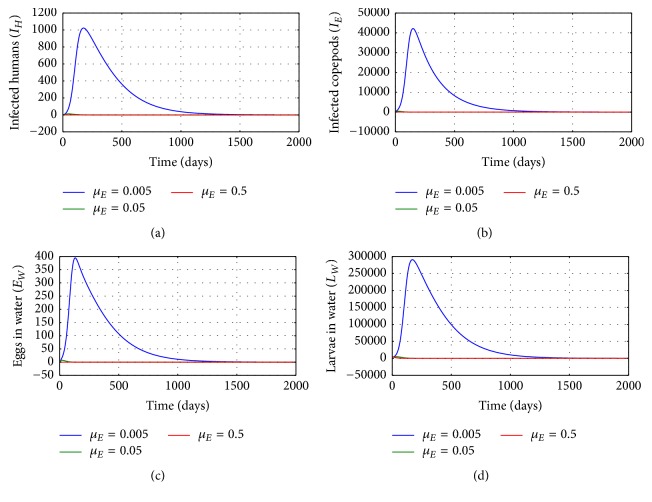
Graphs of numerical solutions of model system (1) showing the evolution with time of (a) population of infected humans (*I*_*H*_), (b) population of infected copepods (*I*_*E*_), (c) population of Guinea worm eggs in the physical water environment, and (d) population of Guinea worm larvae in the physical water environment, for different values of natural death rate of copepods *μ*_*E*_: *μ*_*E*_ = 0.005, *μ*_*E*_ = 0.05, and *μ*_*E*_ = 0.5.

Figure 3 illustrates the solution profile of the population of (a) infected humans, (b) infected copepods in the physical water environment, (c) worm eggs in the physical water environment, and (d) worm larvae in the physical water environment, for different values of natural death rate of copepod population in the physical water environment *μ*_*E*_: *μ*_*E*_ = 0.005, *μ*_*E*_ = 0.05, and *μ*_*E*_ = 0.5. The results show that environmental conditions which increase death of copepods affect transmission of the disease in the human population. Increased death of copepod population reduces transmission risk of the disease at humans population; therefore any mechanisms which enhance the killing of copepod population in the physical water environment reduces transmission risk of GWD within disease endemic communities.

**Figure 4:**
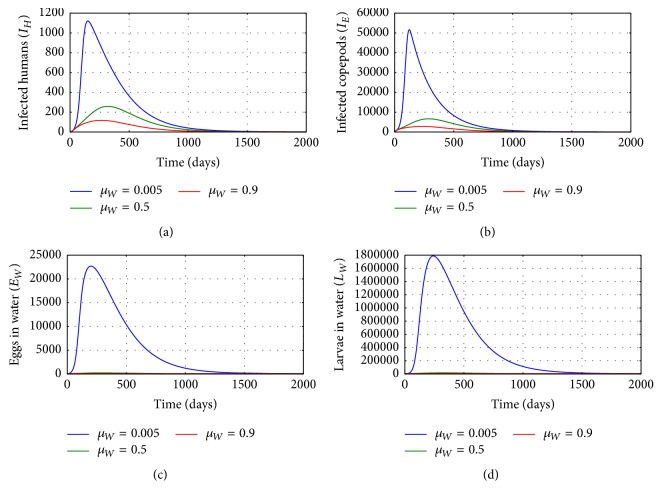
Graphs of numerical solutions of model system (1) showing the evolution with time of (a) population of infected humans (*I*_*H*_), (b) population of infected copepods (*I*_*E*_), (c) population of Guinea worm eggs in the physical water environment, and (d) population of Guinea worm larvae in the physical water environment, for different values of natural death rate of Guinea worm eggs in the physical water environment *μ*_*W*_: *μ*_*W*_ = 0.005, *μ*_*W*_ = 0.5, and *μ*_*W*_ = 0.9.

Figure 4 shows graphs of numerical solutions of model system (1) showing propagation of (a) population of infected humans (*I*_*H*_), (b) population of infected copepods (*I*_*E*_), (c) population of Guinea worm eggs in the physical water environment, and (d) population of Guinea worm larvae in the physical water environment, for different values of natural death rate of Guinea worm eggs in the physical water environment *μ*_*W*_: *μ*_*W*_ = 0.005, *μ*_*W*_ = 0.5, and *μ*_*W*_ = 0.9. The results show that the environmental conditions which enhance death of worm eggs affect transmission of GWD in the human population. Increased death of worm egg population reduces transmission risk of the disease at human population level. Therefore any mechanisms which enhance the killing of worm egg population in the physical water environment reduce transmission risk of the disease within GWD endemic communities.

**Figure 5:**
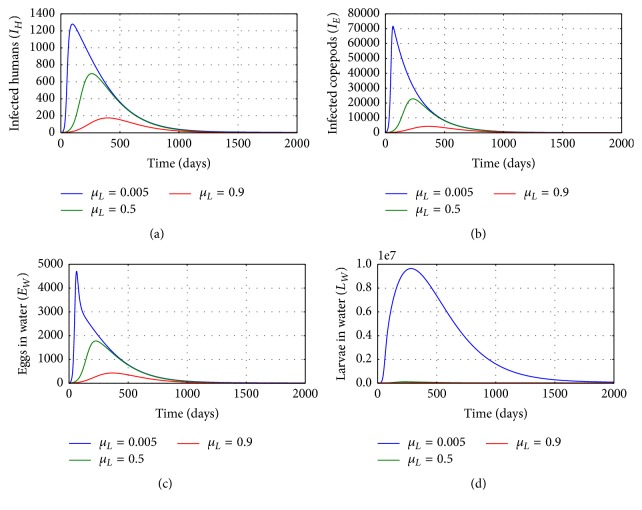
Graphs of numerical solutions of model system (1) showing the evolution with time of (a) population of infected humans (*I*_*H*_), (b) population of infected copepods (*I*_*E*_), (c) population of Guinea worm eggs, and (d) population of Guinea worm larvae in the physical water environment, for different values of natural death rate of Guinea worm larvae in the physical water environment *μ*_*L*_: *μ*_*L*_ = 0.005, *μ*_*L*_ = 0.5, and *μ*_*L*_ = 0.9.

Figure 5 shows graphs of numerical solutions of model system (1) showing propagation of (a) population of infected humans (*I*_*H*_), (b) population of infected copepods (*I*_*E*_), (c) population of Guinea worm eggs, and (d) population of Guinea worm larvae in the physical water environment, for different values of natural death rate of Guinea worm larvae in the physical water environment *μ*_*L*_: *μ*_*L*_ = 0.005, *μ*_*L*_ = 0.5, and *μ*_*L*_ = 0.9. The results show that the environmental conditions which increase death of worm larvae reduce transmission of GWD in the human population. Increased death of worm larvae population reduces transmission risk of the disease at human population level. Therefore any interventions which enhance the killing of worm larvae population in the physical water environment reduce transmission risk of GWD within the human population.

**Figure 6:**
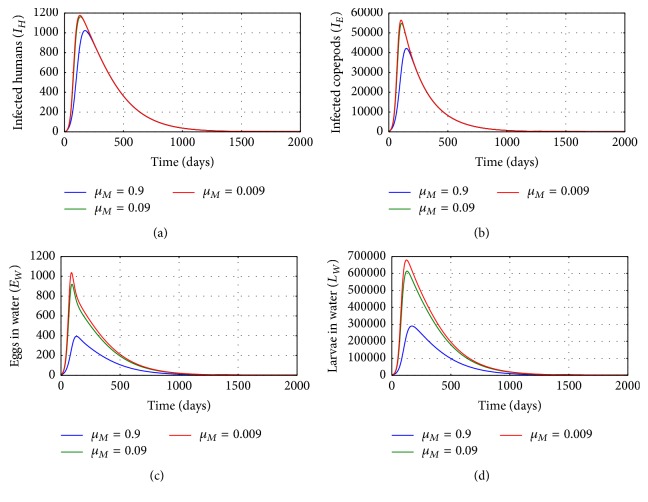
Graphs of numerical solutions of model system (1) showing the evolution with time of (a) population of infected humans (*I*_*H*_), (b) population of infected copepods (*I*_*E*_), (c) population of Guinea worm eggs in the physical water environment, and (d) population of Guinea worm larvae in the physical water environment, for different values of natural death rate of mature worms inside a single infected human host *μ*_*M*_: *μ*_*M*_ = 0.9, *μ*_*M*_ = 0.09, and *μ*_*M*_ = 0.009.

Figure 6 shows graphs of numerical solution of model system (1) showing propagation of (a) population of infected humans (*I*_*H*_), (b) population of infected copepods (*I*_*E*_), (c) population of Guinea worm eggs in the physical water environment, and (d) population of Guinea worm larvae in the physical water environment, for different values of natural death rate of mature worms inside a single infected human host *μ*_*M*_: *μ*_*M*_ = 0.9, *μ*_*M*_ = 0.09, and *μ*_*M*_ = 0.009. The results show that the within-host process of death of mature worms affects transmission of the disease in the human population level. Increased death of mature worm population within an infected human host reduces transmission risk of the disease at human population level. Therefore any interventions which enhance the killing of mature worm population inside an infected human host reduce transmission risk of the disease within communities.

**Figure 7:**
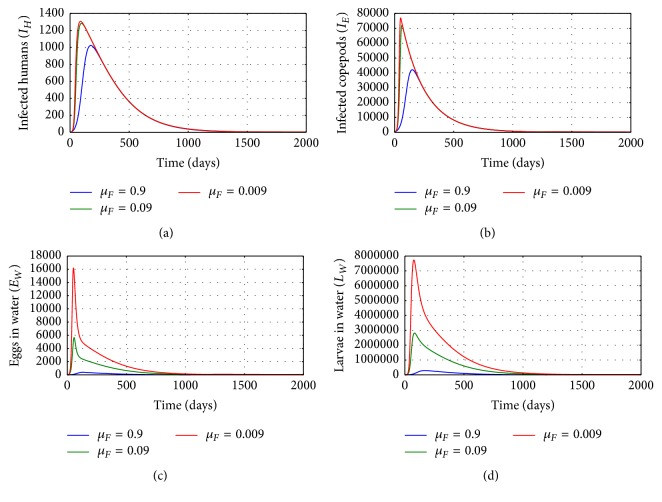
Graphs of numerical solutions of model system (1) showing the evolution with time of (a) population of infected humans (*I*_*H*_), (b) population of infected copepods (*I*_*E*_), (c) population of Guinea worm eggs, and (d) population of Guinea worm larvae in the physical water environment, for different values of natural death rate of fertilized female worm within a single infected human host *μ*_*F*_: *μ*_*F*_ = 0.9, *μ*_*F*_ = 0.09, and *μ*_*F*_ = 0.009.

Figure 7 shows graphs of numerical solution of model system (1) showing propagation of (a) population of infected humans (*I*_*H*_), (b) population of infected copepods (*I*_*E*_), (c) population of Guinea worm eggs, and (d) population of Guinea worm larvae in the physical water environment, for different values of natural death rate of fertilized female worms inside a single infected human host *μ*_*F*_: *μ*_*F*_ = 0.9, *μ*_*F*_ = 0.09, and *μ*_*F*_ = 0.009. The results show that the within-host processes which increase the death of fertilized female worms can be a potent control measure for GWD. Increased death of fertilized female worms within infected human hosts reduces transmission risk of the disease at human population level. Therefore any interventions which enhance the killing of fertilized female worm population inside an infected human host reduce transmission risk of GWD within a community.

**Figure 8:**
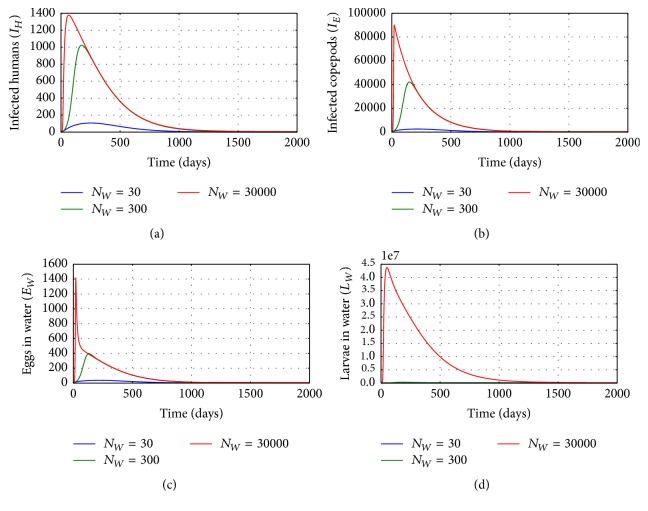
Graphs of numerical solutions of model system (1) showing the evolution with time of (a) population of infected humans (*I*_*H*_), (b) population of infected copepods (*I*_*E*_), (c) population of Guinea worm eggs in the physical water environment, and (d) population of Guinea worm larvae in the physical water environment, for different values of Guinea worm larvae fecundity *N*_*W*_: *N*_*W*_ = 30, *N*_*W*_ = 300, and *N*_*W*_ = 30000.

Figure 8 illustrates the solution profiles of the population of (a) infected humans (*I*_*H*_), (b) infected copepods (*I*_*E*_) in the physical water environment, (c) worm eggs in the physical water environment, and (d) worm larvae in the physical water environment, for different values of the rate of worm larvae fecundity *N*_*W*_: *N*_*W*_ = 30, *N*_*W*_ = 300, and *N*_*W*_ = 30000. The results show that an increase of worm larvae produced per day by worm eggs increases the transmission of the disease. Therefore, any interventions which reduce worm larvae fecundity in the physical water environment reduce the transmission risk of the disease in the community.

**Figure 9:**
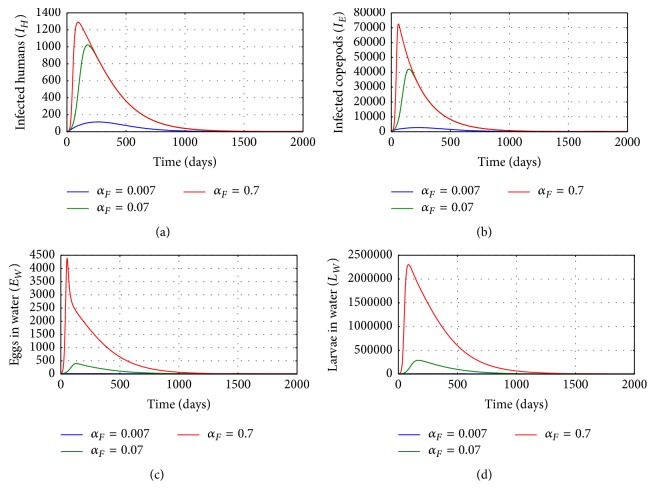
Graphs of numerical solutions of model system (1) showing the evolution with time of (a) population of infected humans (*I*_*H*_), (b) population of infected copepods (*I*_*E*_), (c) population of Guinea worm eggs in the physical water environment, and (d) population of Guinea worm larvae in the physical water environment, for different values of the rate at which an emerging fertilized female worm from a single infected human host excretes eggs into the physical water environment *α*_*F*_: *α*_*F*_ = 0.007, *α*_*F*_ = 0.07, and *α*_*F*_ = 0.7.

Figure 9 shows graphs of numerical solution of model system (1) showing the propagation of the population of (a) infected humans (*I*_*H*_), (b) population of infected copepods (*I*_*E*_), (c) worm eggs in the physical water environment, and (d) worm larvae in the physical water environment, for different values of the rate at which an emerging fertilized female worm from a single infected human host excretes number of eggs into the physical water environment *α*_*F*_: *α*_*F*_ = 0.007, *α*_*F*_ = 0.07, and *α*_*F*_ = 0.7. The results show that higher rate of excretion of worm eggs by each infected human host results in increased population of parasites (worm eggs and worm larvae) in the physical water environment and a noticeable increase in infected copepods. Therefore, improvements in individual sanitation (which reduce contamination of water source with human eggs) are good for the community because they reduce the risk of the disease transmission in the community.

**Figure 10:**
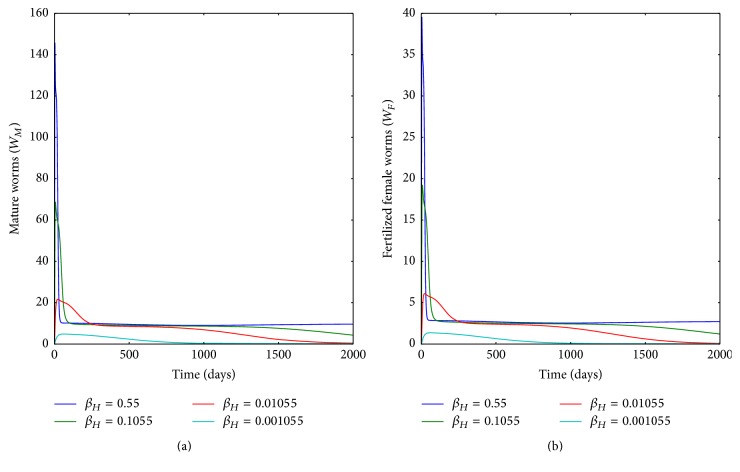
Graphs of numerical solutions of model system (1) showing the evolution with time of (a) population of mature worm within infected human host and (b) population of fertilized female worm within infected human host, for different values of the infection rate of humans *β*_*H*_: *β*_*H*_ = 0.1055, *β*_*H*_ = 0.01055, *β*_*H*_ = 0.001055, and *β*_*H*_ = 0.55.

Figure 10 demonstrates numerical solutions showing the propagation of the population of (a) mature worm within infected human host and (b) population of fertilized female worm within infected human host, for different values of the infection rate of humans *β*_*H*_: *β*_*H*_ = 0.1055, *β*_*H*_ = 0.01055, *β*_*H*_ = 0.001055, and *β*_*H*_ = 0.55. The results show the influence of between-host disease process on within-host disease process of Guinea worm disease. Here, as transmission rate of GWD in the community increases, the within-host infection intensity of the disease also increases. The numerical results demonstrate that the transmission of the disease at the population level influences the dynamics within an infected individual. Therefore, human behavioural changes (such as filtering water before drinking) which reduce contact with infected copepods reduce infection intensity at individual level. Equally, good sanitation by community which reduces contamination of water bodies reduces the intensity of infection of humans at individual level.

**Figure 11:**
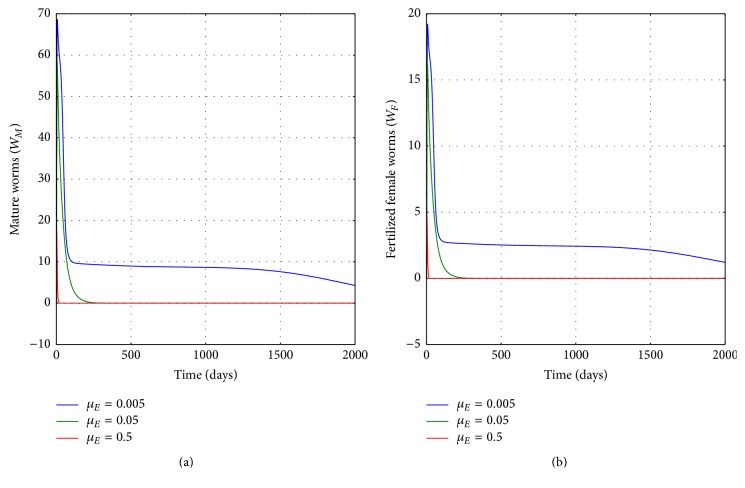
Graphs of numerical solutions of model system (1) showing the evolution with time of (a) population of mature worm within infected human host and (b) population of fertilized female worm within infected human host, for different values of the natural death rate of copepods *μ*_*E*_: *μ*_*E*_ = 0.005, *μ*_*E*_ = 0.05, and *μ*_*E*_ = 0.5.

Figure 11 illustrates the graphs of numerical solutions showing the propagation of the population of (a) mature worm within infected human host and (b) population of fertilized female worm within infected human host, for different values of the natural death rate of copepods in the physical water environment *μ*_*E*_: *μ*_*E*_ = 0.005, *μ*_*E*_ = 0.05, and *μ*_*E*_ = 0.5. The results demonstrate the potency of public health interventions intended to reduce copepods population (such as killing copepods using temephos or boiling the water) on the infection intensity within an infected individual.

**Figure 12:**
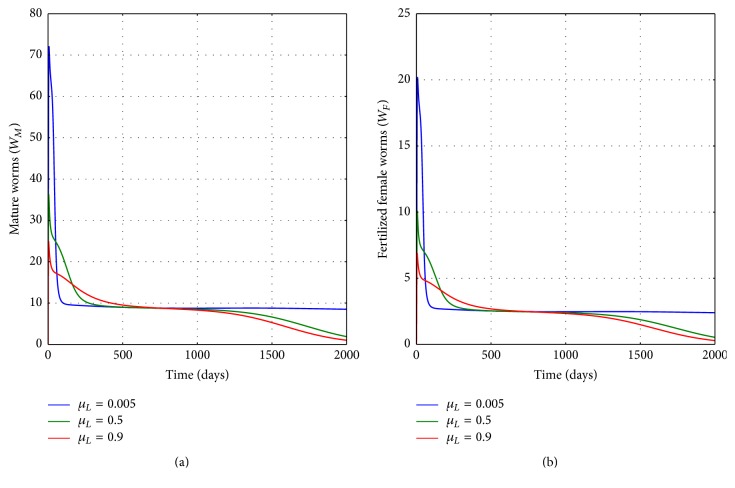
Graphs of numerical solutions of model system (1) showing the evolution with time of (a) population of mature worm within infected human host and (b) population of fertilized female worm within infected human host, for different values of natural death rate of worm larvae in the physical water environment *μ*_*L*_: *μ*_*L*_ = 0.005, *μ*_*L*_ = 0.05, and *μ*_*L*_ = 0.5.

Figure 12 illustrates the graphs of numerical solutions showing the propagation of the population of (a) mature worm within infected human host and (b) population of fertilized female worm within infected human host, for different values of the natural death rate of worm larvae in the physical water environment *μ*_*L*_: *μ*_*L*_ = 0.005, *μ*_*L*_ = 0.05, and *μ*_*L*_ = 0.5. The results demonstrate the influence of public health interventions intended to reduce worm larvae population in the physical water environment (such as destroying worm larvae using chemicals or boiling the water) on the infection intensity within an infected individual. Overall, the numerical results verify the following aspects about GWD transmission dynamics.

- Higher rates of infection at the human population level result in increased population of parasites (worm eggs and worm larvae) in the physical water environment and a noticeable increase in infected copepod population in the physical water environment.
- Interventions which increase death of copepods through enhanced killing of copepod population in the physical water environment reduce transmission risk of GWD within a disease endemic communities.
- Interventions which enhance death of worm eggs through enhanced killing of worm egg population in the physical water environment reduce transmission risk of the disease within GWD endemic communities.
- Health interventions which increase death of worm larvae in the physical water environment reduce transmission risk of GWD within the human population.
- Within-host scale interventions which increase death of mature worm population inside an infected human host reduce transmission risk of the disease within communities.
- Within-host scale interventions which increase the death of fertilized female worms can also be a potent control measure for GWD through reduced transmission risk of GWD within a community.
- An increase in worm fecundity with an infected human host has considerable impact on the transmission of GWD.
- Higher rate of contamination of the physical water environment through excretion of worm eggs by each infected human host results in increased population of parasites (worm eggs and worm larvae) in the physical water environment and a noticeable increase in infected copepods.
- Transmission of the GWD shows reciprocal influence of within-host scale interventions (medical interventions) and between-host scale interventions (public health interventions). Therefore, human behavioural changes (such as filtering water before drinking) which reduce contact with infected copepods reduce infection intensity at individual level. Equally, good sanitation by community which reduces contamination of water bodies reduces the intensity of infection of humans at individual level.

## 8. Conclusions

Guinea worm disease, like most neglected parasitic diseases, urgently needs renewed attention and sustainable interventions in Africa. The limited scientific knowledge about GWD represents a challenge to the successful elimination of the disease. In this paper, we have sought to identify a broad range of within-host and between-host processes that should be better understood if GWD is to be eliminated. A multiscale model of GWD transmission dynamics is presented. The multiscale model is shown to be mathematically and epidemiologically well-posed. Sensitivity analyses of the basic reproduction number to the variation of model parameters were carried out. The sensitivity results of the reproduction number show that between-host model parameters (such as infection rate of human host *β*_*H*_ and supply rate of humans Λ_*H*_); within-host model parameters (such as excretion rate of eggs *α*_*F*_ into the physical water environment by each infected human host, fecundity rate of mature worm *N*_*C*_, decay rate of mature worms *μ*_*M*_, migration rate of mature worms to the subcutaneous tissue *α*_*M*_, and decay rate of fertilized worms *μ*_*F*_); and environmental model parameters (such as the production rate of larvae per egg worm per day *α*_*W*_, fecundity of larvae *N*_*W*_ generated by eggs, death rate of egg worms *μ*_*W*_, larva worms *μ*_*L*_ in the physical water environment, supply rate of copepods Λ_*E*_, and decay rate of copepods *μ*_*E*_) all are responsible for the transmission dynamics of Guinea worm disease within the community. Therefore reducing the infection rate of human, excretion rate of eggs into physical water, and the population of parasites (worm eggs and worm larvae) as well as population of vector host (copepods) in the physical water environment could eventually contribute in eradicating GWD completely from the community.

## Figures and Tables

**Table 1:** Description of the state variables of the model system (1).

State variable	Description	Initial value
*S*_*H*_(*t*)	The susceptible human population size in the behavioural human environment	2500
*I*_*H*_(*t*)	The infected human population size in the behavioural human environment	10
*I*_*C*_(*t*)	The infected copepod population size in the biological human environment	0
*W*_*M*_(*t*)	The mature worm population size in the biological human environment	0
*W*_*F*_(*t*)	The female worm population size in the biological human environment	0
*G*_*J*_(*t*)	Amount of gastric acid in the human stomach	1.5
*S*_*E*_(*t*)	The susceptible copepod population size in the physical water environment	10^5^
*I*_*E*_(*t*)	The infected copepod population size in the physical water environment	0
*E*_*W*_(*t*)	The worm egg population size in the physical water environment	0
*L*_*W*_(*t*)	The worm larvae population size in the physical water environment	5000

**Table 2:** Number of possible positive roots of *h*(*I*_*E*_^*∗*^) = 0.

Cases	*γ*_3_	*γ*_2_	*γ*_1_	*γ*_0_	Number of sign changes	Number of possible real roots (endemic equilibrium)
1	+	+	+	+	0	0
2	+	+	+	−	1	1
3	+	+	−	+	2	0, 2
4	+	+	−	−	1	1
5	+	−	−	+	2	0, 2
6	+	−	−	−	1	1
7	+	−	+	+	2	0, 2
8	+	−	+	−	3	1, 3

**Table 3:** Sensitivity indices of model reproduction number *R*_0_ to parameters for model system (1), evaluated at the parameters values presented in Tables 4–6.

Parameter	Description	Sensitivity index with positive sign	Sensitivity index with negative sign
*μ*_*E*_	Natural decay rate of copepods in the water environment		−0.9991
*α*_*C*_	Natural decay rate of copepods within human host		−0.4853
*μ*_*C*_	Release rate of mature worms within human host		−0.4853
Λ_*H*_	Human birth rate	+0.50013	
*β*_*H*_	Human infection rate	+0.5	
*N*_*W*_	Fecundity rate of worm larvae in the environment	+0.5	
*μ*_*L*_	Natural decay rate of Guinea worm larvae		−0.5
*L*_0_	Larvae saturation constant		−0.5
*N*_*C*_	Fecundity rate of mature worm	+0.5	
Λ_*E*_	Copepods birth rate	+0.5	
*β*_*E*_	Copepods infection rate	+0.5	
*P*_0_	Copepods saturated constant		−0.5
*α*_*H*_	Human recovery		−0.4998
*μ*_*F*_	Natural decay rate of fertilized female worms		−0.4639
*α*_*F*_	Migration rate of fertilized female worms to surface of host's skin	+0.4639	
*μ*_*W*_	Natural decay rate of worm eggs in the water environment		−0.4545
*α*_*W*_	Worm egg hatching rate	+0.4545	
*μ*_*M*_	Natural decay rate of mature worms within human host		−0.25
*α*_*M*_	Migration rate of mature worms to subcutaneous tissues	+0.25	
*μ*_*J*_	Dilution/degradation rate of gastric juice		−0.0147
*G*_0_	Supply rate of gastric juice from the source of the body	+0.0147	
*δ*_*E*_	Induced decay rate of copepods in the water environment		−0.000894

**Table 4:** Human host parameter values used in simulations.

Parameter	Description	Initial values	Units	Source
Λ_*H*_	Human birth rate	0.1013	People day^−1^	[14]
*β*_*H*_	Human infection rate	0.1055	Copepod day^−1^	Estimated
*μ*_*H*_	Human natural death rate	2.548 × 10^−5^	Day^−1^	[15, 16]
*α*_*H*_	Human recovery rate	0.03	Day^−1^	Estimated
*δ*_*H*_	GWD induced death rate	4 × 10^−8^	Day^−1^	Estimated

**Table 5:** Within-host parameter values of the model system (1).

Parameter	Description	Initial values	Units	Source
*N*_*C*_	Fecundity rate of mature worms	700	People	Estimated
*μ*_*C*_	Decay rate of copepods within a human host due to gastric juice	0.99	Copepod day^−1^	Estimated
*α*_*C*_	Natural death rate of copepods within a human host	0.001	Day^−1^	Estimated
*μ*_*M*_	Natural decay rate of mature worms within a human host	0.9	Day^−1^	Estimated
*α*_*M*_	Migration rate of mature worms to subcutaneous tissues	0.9	Day^−1^	Estimated
*μ*_*F*_	Natural death rate of fertilized female worms within a human host	0.9	Day^−1^	Estimated
*α*_*F*_	Migration rate of fertilized female worms to surface of skin	0.07	Day^−1^	Estimated
*μ*_*J*_	Dilution/degradation rate of gastric juice	0.05	Day^−1^	Estimated
*α*_*J*_	Proliferation rate of gastric juice due to infection	0.4	Day^−1^	Estimated
*G*_0_	Supply rate of gastric juice from within a human body	1.5	Day^−1^	Estimated

**Table 6:** Free-living pathogens and their associated environmental parameter values used in simulations.

Parameter	Description	Initial value	Units	Source
Λ_*E*_	Copepods birth rate	0.75	Copepod day^−1^	Estimated
*β*_*E*_	Copepods infection rate	0.7	Larvae day^−1^	Estimated
*μ*_*E*_	Natural decay rate of copepods	0.005	Day^−1^	[15, 16]
*δ*_*E*_	Disease induced death rate of copepods	9 × 10^−6^	Day^−1^	Estimated
*P*_0_	Copepods saturation constant	20 0000	Day^−1^	[15, 16]
*μ*_*W*_	Natural decay rate of Guinea worm eggs	0.333	Day^−1^	[15, 16]
*α*_*W*_	Hatching rate of worm eggs	0.009	Day^−1^	Estimated
*N*_*W*_	Number of Guinea worm larvae hatched	300	Larvae egg ^−1^ day^−1^	Estimated
*μ*_*L*_	Natural decay rate of Guinea worm larvae	0.0333	Day^−1^	[15, 16]
*L*_0_	Larvae saturation constant	5000000	Day^−1^	[15, 16]
*ϵ*	Limitation growth rate	0.0991	Day^−1^	Estimated
